# Microbiological and genomic approaches to study and characterize co- and cross-resistance to antimicrobial peptides in *Listeria monocytogenes* ATCC 19,111 variants

**DOI:** 10.1186/s12866-026-05467-w

**Published:** 2026-08-07

**Authors:** Omar Fliss, Louis-David Guay, Nguyen Phuong Pham, Philippe Leprohon, Ramzi Guerbaa, Marc Ouellette, Natalie Fava, Franco Pagotto, Éric Biron, Ismail Fliss

**Affiliations:** 1 Département des Sciences des Aliments, Faculté des Sciences de l’agriculture et de l’alimentation, Université Laval, Québec (QC), Canada; 2 Centre de Recherche du CHU de Québec-Université Laval, Québec (QC), Canada; 3 Research Center in Infectious Diseases, Université Laval, Québec (QC), Canada; 4 Institut of Nutrition and Functional Foods, Université Laval, Québec (QC), Canada; 5 Faculté de Pharmacie, The Quebec Network for Research on Protein Function, Engineering, and Applications, Université Laval and PROTEO, Québec (QC), Canada; 6 Département de microbiologie, infectiologie et immunologie, Faculté de médecine, Université Laval, Québec (QC), Canada; 7 Listeriosis Reference Centre, Food Safety Regulatory Research Division, Bureau of Microbial and Toxicological Food Safety, Food and Nutrition Directorate, Health Canada, Ottawa, Canada

**Keywords:** Antimicrobial peptides, Antimicrobial resistance, Co-resistance, Cross-resistance, Genomic analysis, *Listeria monocytogenes*, Metabolic analysis, Whole-genome sequencing

## Abstract

**Background:**

Antimicrobial peptides are considered a promising alternative to conventional antibiotics for controlling *Listeria monocytogenes*, a major foodborne pathogen. However, the emergence of antimicrobial resistance, including co- and cross-resistance between structurally and functionally distinct peptides, remains a critical and insufficiently explored challenge. This study aimed to investigate and characterize the mechanisms underlying co- and cross-resistance among five distinct antimicrobial peptides: nisin Z, pediocin PA-1, plantaricin S, brevibacillin, and its synthetic analog brevibacillin Thr1, selected for their diverse structures and modes of action.

**Results:**

All tested peptides exhibited strong anti-listerial activity, with minimum inhibitory concentrations ranging from 0.5 to 15 µM and minimum bactericidal concentrations from 1 to 60 µM. Resistant variants were generated through stepwise adaptive evolution by exposing *Listeria monocytogenes* ATCC 19,111 to gradually increasing peptide concentrations. These variants were characterized based on resistance levels, growth rates, and resistance stability. No cross-resistance was observed between antimicrobial peptides and conventional antibiotics. However, co-resistance and cross-resistance were identified among certain peptides, particularly those sharing structural or functional similarities, suggesting overlapping or convergent resistance mechanisms. Whole-genome sequencing and metabolic analyses revealed distinct genetic mutations associated with resistance phenotypes, along with specific alterations in metabolic pathways.

**Conclusion:**

This study demonstrates that *Listeria monocytogenes* ATCC 19,111 can develop co- and cross-resistance to antimicrobial peptides, especially among structurally or functionally related compounds. The findings highlight the complexity of resistance mechanisms and emphasize the importance of integrating genomic and metabolic approaches to better understand adaptive responses to antimicrobial pressure. These insights may inform the development of more effective antimicrobial strategies and support the rational design of peptide-based interventions to control foodborne pathogens.

**Supplementary Information:**

The online version contains supplementary material available at https://doi.org/10.1186/s12866-026-05467-w.

## Introduction

*Listeria monocytogenes* is a food-borne pathogen associated with significant public health risks, particularly for vulnerable populations such as pregnant women, newborns, the elderly and the immunocompromised [1, 2]. Listeriosis outbreaks are frequently associated with the consumption of contaminated food, underlining the urgent necessity for effective strategies to control this pathogen in the food industry [3]. Furthermore, the increased demand for environmentally friendly and natural food additives has generated a growing need to investigate innovative alternative strategies for food preservation [4].

For decades, antimicrobial peptides (AMPs) such as bacteriocins (ribosomally synthesized peptides) and lipopeptides (non-ribosomally synthesized peptides) have emerged as potential natural preservatives [5]. Among them, pediocin PA-1, plantaricin S and nisin Z are examples of bacteriocins produced by lactic acid bacteria (LAB) that have displayed significant anti-*Listeria* activity [6–8]. These molecules exhibit diverse physicochemical properties and mechanisms of action, mainly involving membrane disruption, receptor interaction and alteration of cellular homeostasis, which contribute to their broad antimicrobial activity [9, 10]. These compounds exhibit distinct mechanisms of action, mainly targeting the bacterial membrane, while being active against a wide range of bacteria, including multidrug-resistant bacterial (MDR) strains [11]. In addition to their biological and technological properties, bacteriocins and lipopeptides demonstrate significant potential to be used as biocontrol agents, natural preservatives or adjuvants in the treatment of infections, particularly in the medical, veterinary and agrifood sectors [5, 12].

Despite their growing popularity, the widespread use of these AMPs in various sectors remains relatively limited. As with antibiotics, microorganisms can develop resistance to bacteriocins and lipopeptides, thus limiting their uses. Indeed, repeated exposure to antimicrobial compounds creates a selective pressure that can favor the emergence of adapted variants through genetic modifications and physiological adjustments improving bacterial survival [13]. These adaptive responses may involve alterations in membrane composition, cell wall structure, regulatory networks, transport systems and metabolic pathways. Unlike antibiotics, however, resistance to different classes of AMPs remains a poorly characterized phenomenon. Moreover, the definition of bacteriocin resistance is not yet clear in the literature. Acquired resistance can result from many mechanisms, including spontaneous mutation of genes involved, for example, in receptor expression, cell wall synthesis, transcription regulators, as well as energy metabolism and transport [14]. Therefore, the investigation of resistance to AMPs is an essential component in studying their activity and for their use [15]. Furthermore, the identification of environmental and physiological factors influencing the emergence of these variants is a major challenge [16]. These insights are essential for establishing optimal strategies to ensure antimicrobial efficacy in food applications, while simultaneously minimizing the risk of the emergence of resistant variants [16]. Previous research has investigated the modes of action and resistance of several antimicrobial agents, providing key insights regarding the intrinsic mechanisms of resistance and acquired resistance following repeated exposure to numerous antimicrobial agents [16–18]. For example, the resistance mechanism of *L. monocytogenes* to nisin Z has been generally associated with a modification in the membrane fatty acid composition [13]. Resistance to pediocin PA-1 was associated with the modification in a specific membrane receptor, the mannose phosphotransferase [17]. However, most previous studies have focused on individual AMP–bacterium interactions, and limited information is available regarding whether adaptation to one AMP can influence susceptibility to other AMPs or conventional antibiotics. In this context, two major phenomena, namely cross-resistance and co-resistance require further investigation. Cross-resistance refers to the acquisition of resistance to multiple antimicrobial compounds following adaptation to a single selective pressure, usually through shared cellular mechanisms or overlapping stress responses [19]. Co-resistance refers to the simultaneous presence or acquisition of resistance traits against different antimicrobial compounds, which may arise from common genetic determinants or coordinated physiological adaptations [19]. Understanding these phenomena is particularly important for assessing the potential risks associated with the combined or sequential use of AMPs and conventional antimicrobial agents. Despite these observations, data on resistance mechanisms remain limited, particularly regarding clinically or food-related pathogenic bacteria. This knowledge gap impedes the assessment of resistance risk development when these compounds are used in combination or in clinical or agrifood contexts.

Recently, we have successfully produced and characterized brevibacillin as well as several analogues, a lipopeptide naturally produced by numerous *Bacillus* spp strains [20]. We also showed a significant inhibition activity against several foodborne pathogens, including *L. monocytogenes* [21]. Among these analogues, brevibacillin Thr1 was developed through a targeted structural modification of the native brevibacillin sequence, generating a synthetic variant with modified physicochemical properties while maintaining the lipopeptide characteristics of the parental molecule. Therefore, in the present study, brevibacillin Thr1 was not considered as an independent AMP class, but rather as a structurally related analogue of brevibacillin, allowing the evaluation of the impact of a specific structural modification on antimicrobial activity, bacterial adaptation as well as resistance development. Despite the fact that brevibacillin has shown promising antimicrobial activity, its molecular resistance mechanisms remain largely unexplored. This may be explained in part by the lack of information about the intracellular targets in sensitive bacteria as well as the adaptive responses they trigger among the targeted pathogens. Moreover, the mechanisms by which bacteria simultaneously undergo genetic and metabolic adaptation following AMPs exposure remain poorly understood.

In addition, beyond the individual characterization of resistance mechanisms to each peptide, co- and cross-resistance is a topic that remains largely unexplored. In particular, the potential cross-resistance between antimicrobial peptides and conventional antibiotics from various classes is still not well investigated [22]. Similarly, co-resistance between AMPs resulting from simultaneous or subsequent selective pressures has emerged as a growing concern and remains insufficiently documented [23]. A deeper understanding and characterization of these phenomena is essential not only to assess the risk of cross-resistance between different peptides or between peptides and antibiotics, as well as co-resistance between different AMPs, but also to establish a solid scientific basis for the combined use of antimicrobial molecules, therefore reducing the risk of resistance development in various applications. The microbial resistance mechanisms are usually investigated through the identification of specific genetic determinants, including specific gene mutations, as well as the detection of genetic mobile elements, such as plasmids, transposons or integrons, enabling the horizontal dissemination of these traits [24, 25]. This genetics-based approach has allowed the understanding of numerous molecular aspects underlying the acquired resistance to several AMPs [26]. However, this approach alone cannot adequately provide insights into several complex phenotypes, such as those associated with cross-resistance between different AMPs classes or between AMP and conventional antibiotics. In this context, the importance of integrating metabolic approaches has emerged as a complementary and promising research avenue [27]. Indeed, the metabolic adjustments induced by the selective pressure of antimicrobials can highlight crucial physiological adaptations, occasionally unrelated to known genetic factors, and provide relevant information about bacterial adaptation and survival strategies [27, 28]. In addition to the conventional phenotypic methodology, a combination of genetic and metabolic approaches would allow a better understanding of the resistance mechanisms, as well as the identification of possible causality relationships between specific mutations and altered metabolic profiles [29]. This approach could be particularly relevant for elucidating the complex mechanisms of co- and cross-resistance between various AMPs from different classes, as well as between AMPs and antibiotics.

In this context, the present study focused on assessing the antimicrobial activity and resistance profile of five AMPs belonging to different classes and having distinct mechanisms of action. Three bacteriocins, nisin Z (class I, lantibiotic), pediocin PA-1 (class IIa) and plantaricin S (class IIb) and two lipopeptides, brevibacillin and its synthetic analog brevibacillin Thr1, were investigated as a peptide pair to determine how structural modification may influence susceptibility patterns and resistance development. These peptides screened for their inhibitory activity against a collection of *Listeria monocytogenes* isolates from food and clinical origins. This study also aimed to characterize the resistance profiles and investigate potential co- and cross-resistance, between the different AMPs as well as between AMPs and antibiotics. Furthermore, this study aimed to determine how AMP selective pressure drives bacterial adaptation and to identify the associated genomic and metabolic changes underlying the emergence of resistant variants. An integrative approach combining phenotypic, genomic and metabolic analyses was used to provide a deeper insight into the underlying resistance mechanisms.

## Methods

### Bacterial strains and culture conditions

Twenty *L. monocytogenes* isolates were kindly provided by the Health Public Branch (HPB) and the Canadian Reference Centre for Listeriosis, 251 Sir Frederick Banting Drive, Ottawa, Ontario. Cultures were performed under aerobic conditions for 18 h at 37 °C in tryptic soy broth (TSB) medium (Difco Laboratories, Sparks, MD, USA). All Listeria isolates were preserved in glycerol stocks (20% glycerol) using cryopreservation tubes held at -80 °C. Before each experiment, isolates were subcultured under the appropriate growth conditions, and the final cultures were prepared 24 h prior to experimental use.

### Antimicrobial peptides production and quantification

Five AMPs were selected and used in this study. Pediocin PA-1 (M31L), plantaricin S, brevibacillin and its analog were produced by Fmoc-based solid-phase peptide synthesis (SPPS) on a Liberty Blue 2.0 microwave peptide synthesizer (CEM, Matthews, NC, USA) as previously described [30–32]. Nisin Z was purified by repeated centrifugation steps using Niseen^®^-Siveele, a commercial formulation [33]. A reversed-phase chromatography system using a Shimadzu Prominence system was employed to purify all peptides, with the exception of nisin Z, on a Phenomenex Kinetex^®^ EVO C18 preparative column (250 × 21.2 mm, 300 Å, 5 μm,14 ml/min). Component chromatographic separation was carried out at a flow rate of 14 mL/min using a gradient consisting of 0.1% AcOH/H₂O (A) and 0.1% AcOH/CH₃CN (B) for the purification of pediocin PA-1 (M31L), plantaricin S. For brevibacillin and its analog, a linear gradient separation from 10 to 60% phase B was performed over 20 min at a flow rate of 12 mL/min. Mobile phases consisted of 0.1% TFA/H₂O (A) and 0.1% TFA/CH₃CN (B), using UV detection at 220 and 254 nm. Then, the pure fractions were recovered and subsequently analysed using a Shimadzu Prominence liquid chromatography-mass spectrometry (LC-MS) system equipped with an electrospray ionisation (ESI) probe, using a Kinetex^®^ column (4.6 mm × 100 mm, 2.6 μm XB-C18, 100 Å, 1.8 ml/min) to confirm the purity and identity of each molecule. The characterized peptides were lyophilized using a Speedvac SC250EXP concentrator (ThermoFisher Scientific, Waltham, MA, US).

### AMPs-resistant variants generation

**Fig. 1:**
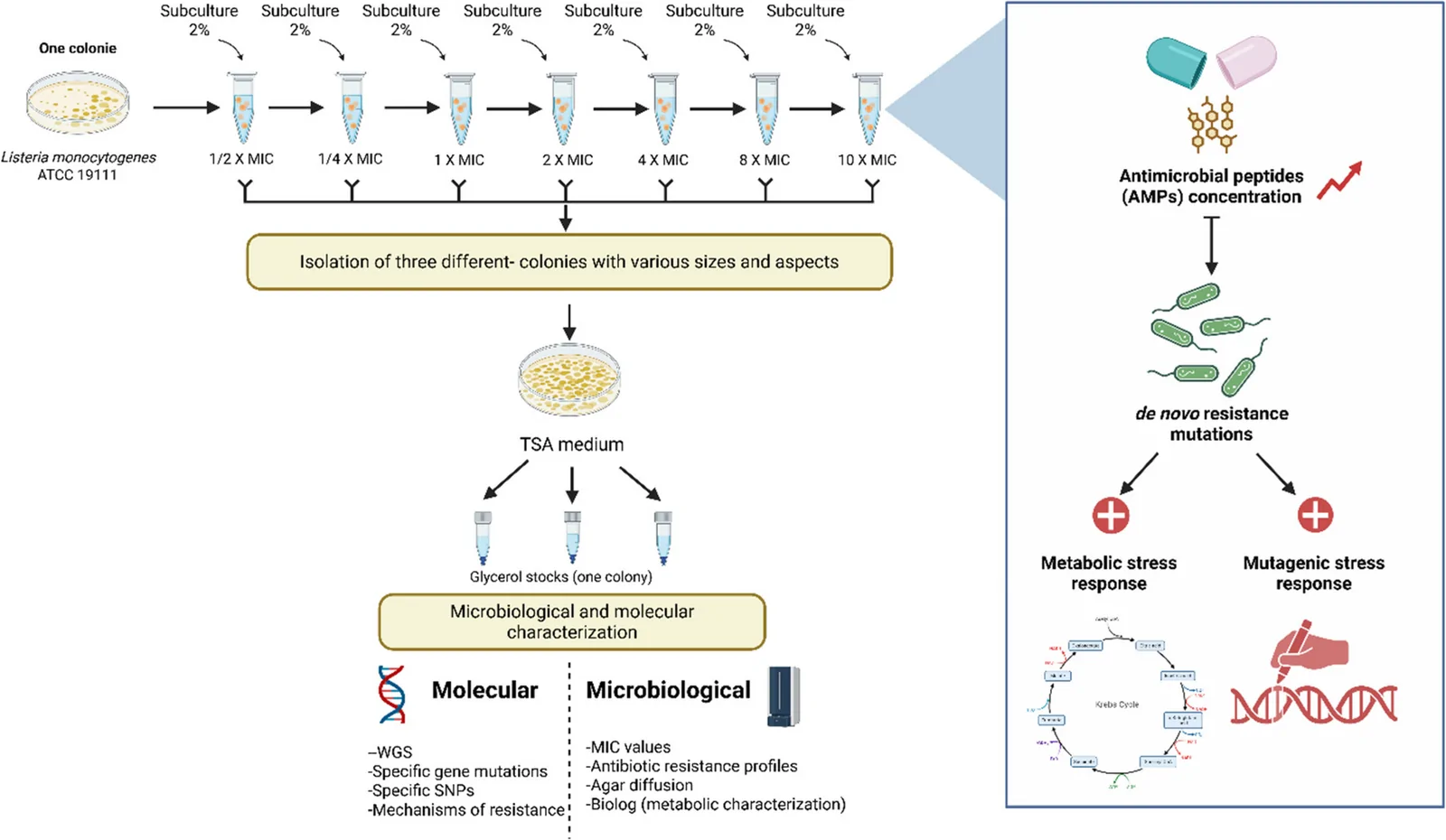
Sequential exposure to antimicrobial peptides and clonal isolation procedure for *Listeria monocytogenes* ATCC 19,111 under selective pressure (Created with BioRender)

The adapted resistant variants to different classes of antimicrobial peptides were generated through a stepwise adaptive evolution approach (Fig. 1). Briefly, an overnight *Listeria monocytogenes* ATCC 19,111 culture was adjusted to cell initial density of approximately 10⁶ CFU/mL, then successively exposed to increasing doses of each peptide from one-quarter, one-half, one-fold, two-fold, four-fold, eight-fold and ten-fold of their MIC, with an inter-culture interval of 24 h [34]. For each step, the cultures were inoculated at 2% (V/V) and incubated with shaking (150 rpm) at 37 °C in TSB medium. Three independent evolutionary lineages were maintained in parallel for each antimicrobial peptide throughout the selection procedure to ensure independent adaptation events. At the end of each incubation period, the surviving bacterial populations showing growth under the selective peptide concentration were transferred to fresh TSB medium containing the next peptide concentration. Growth was assessed by comparison with the corresponding non-selective control cultures before progression to the next selection step. Upon adaptation to the highest tested concentration, the obtained resistant variants were stored as glycerol stocks at -80 °C for further investigations. The stability of the acquired resistance phenotype was subsequently evaluated before whole-genome sequencing and downstream phenotypic analyses to confirm the persistence of the adapted phenotype beyond the initial selection pressure. The three independent AMP-adapted variants obtained for each peptide were subjected to whole-genome sequencing to identify genetic changes potentially associated with the resistance phenotype. Biological assays were performed in duplicate using the three adapted variants exhibiting the highest resistance levels, as determined by their increased MIC values.

### Monitoring the growth kinetics and curves analysis

The different adapted variants and the wild-type strain were cultivated in TSB medium at 37 °C until reaching the mid-log phase. Microbial concentration was then adjusted to approximately 10^4^ CFU by optical density (OD) measurement at 600 nm. To evaluate stable physiological changes associated with the acquired AMP-resistant phenotype, rather than the transient response to antimicrobial exposure, all growth kinetics assays were performed under identical AMP-free conditions, allowing direct comparison between the resistant variants and the parental strain. The Biolog system (Hayward, California, United States) was used for monitoring microbial growth kinetics [35]. Briefly, the 96-well Biolog microplates (Biolog, USA) were inoculated with 100 µL of TSB containing 1% (V/V) Biolog redox H dye mixture. Following this, 50 µL of each adjusted culture was inoculated separately into each well. Control wells were included for samples containing only bacteria without redox reagent (dye mix H) and samples containing only TSB with H dye mixture. The prepared microplates were loaded into the Biolog system and incubated at 37 °C for 24 to 48 h with colour change measurements conducted every 15 min. The specific growth rates (h) of the AMPs resistant variants were compared with those of the wild-type strain. The analysis focused on the growth dynamics, including generation time, growth rate and the cell density. Results were illustrated using kinetic growth curves and statistical comparisons were performed to assess the impact of AMP-induced mutations on bacterial growth parameters.

### Antimicrobial assays

The susceptibility to different AMPs of *L. monocytogenes* wild-type strain and AMP-resistant variants was assessed qualitatively and quantitatively by determining the minimum inhibitory concentration (MIC) and minimum bactericidal concentration (MBC) using agar diffusion and microdilution assays as described by Fliss et *al* [21]. For these experiments, six antibiotics were tested, namely, ampicillin (AM), chloramphenicol (CHL), gentamicin (GM), kanamycin (KAN), tetracycline (TET) and vancomycin (VA). Subsequently, 50 µL of bacterial inoculum, diluted to approximately 10^4^ CFU per well, was added, followed by incubation at 37 °C in TSB broth supplemented with 1% (V/V) H dye mixture reagent for 24 h [36]. The Biolog system was used as a metabolic activity monitoring platform for microdilution assays, where bacterial growth was indirectly assessed through the reduction of the redox dye contained in Dye Mix H. Therefore, the measured signal reflects cellular metabolic activity rather than direct optical density (OD) measurement. A negative control was established using the dye mix with TSB and 10% DMSO, while the positive control included the indicator strain without AMPs exposure versus the negative control. The MIC was defined as the lowest peptide concentration resulting in the absence of detectable metabolic activity compared with the negative control. The MBC was assessed by extending the incubation of the same microdilution plates up to 48 h to determine any variations between the 24 h and 48 h values obtained. The MBC is defined as the lowest concentration that completely inhibits the growth of the indicator strain in the wells. Both the MIC and MBC experiments were conducted in duplicate. MIC and MBC values were determined from the metabolic activity profiles obtained using the Biolog system after 24 h and 48 h of incubation, respectively.

The antimicrobial effect of each peptide was determined by calculating the R ratio, defined as the ratio between MIC and MBC concentration [37, 38]. A bactericidal effect is characterized when the R ratio is ≤ 4, indicating that the peptide has the capacity to effectively destroy bacteria at concentrations close to that required to inhibit their growth. However, a bacteriostatic effect is characterized by an R ratio > 4, suggesting that the peptide is inhibiting bacterial growth without totally eliminating them at the tested concentrations. This classification was based on the experimentally determined MBC/MIC ratios.

### Biolog high-throughput phenotypic microarray assay

The phenotypic microarray (PM) assay was performed using the Biolog system [35, 39]. The assay included four PM panels, PM1 and PM2, specifically selected to evaluate the adapted resistant variants ability to metabolize 190 carbon sources. In addition, panels PM9 and PM10 were used to assess the response of various ionic, osmotic and pH variations. Firstly, PM’s specific additive solutions were prepared in accordance with the standard Biolog. Inc protocols [40]. Secondly, all selected isolates were individually seeded on Tryptic Soya Agar (TSA) medium (Difco Laboratories, Sparks, MD, USA) and incubated at 37 °C for 18-20 h. The adapted variants were grown under AMP-free conditions prior to PM analysis to characterize their intrinsic metabolic phenotype and identify stable metabolic alterations associated with AMP adaptation rather than transient metabolic responses induced by acute peptide exposure. Starting from this culture, a single colony was isolated, and cell suspensions were prepared using a sterile swab. This suspension was diluted in inoculation fluid, respectively: IF-0a GN/GP (20 mL) for PM1 and PM2 plates, and IF-10b GN/GP (110 mL) for PM9 and PM10 plates. The inoculum preparation and turbidity standardization were performed according to the manufacturer’s recommendations for the Biolog Phenotype Microarray system. Briefly, bacterial cells were transferred into the appropriate inoculation fluid supplemented with Dye Mix H and the corresponding PM-specific additive solutions. The cell density was initially adjusted to a turbidity level of 81% according to the Biolog protocol. The suspension was then homogenized, and the transmittance (T) value was adjusted to 50% using the turbidimeter, corresponding to a final standardized inoculum density of 95%, as recommended by Biolog Inc. Subsequently, 100 µL of the previously standardized cell suspensions was added to each well. Plates were then placed in the Biolog reader and incubated at 37 °C for 48 h. Data were recorded every 15 min, converting the pixel density in each well into a signal value reflecting cell growth and dye conversion rather than a direct absorbance measurement. The phenotypic data from the microarray were then analyzed using the signal value calculation method [41]. For this study, *L.monocytogenes* ATCC 19,111 was selected as a reference strain, and substrates were tested in duplicate.

### Whole genome sequencing and genomic analysis

The DNA from the reference strain and its various resistant variants was extracted using the QIAGEN DNeasy Blood and Tissue Kit, in accordance with the manufacturer’s protocol specifically designed for Gram-positive bacteria. Two independent colonies from different resistant variants were separately selected and isolated after each exposure step, gradually increasing starting from a concentration corresponding to one-fold the MIC value, to the highest concentration allowing growth for each mutant. For each mutant, three independent trials and three generations were carried out. Retrieved DNA was then sequenced using the Illumina whole genome sequencing (WGS) platform hosted Laval University Hospital Center (CHUL), Quebec, Canada. Library size distribution and quantification were performed using the QuantiFluor dsDNA system. Paired-end sequencing was carried out on the NovaSeq 6000 sequencing system. Following this, sequencing reads were trimmed for base quality using Trimmomatic and then assessed for quality using FASTQ: https://www.bioinformatics.babraham.ac.uk/projects/fastqc/ [42]. The draft genomes of the selected isolates were assembled de novo using SPAdes version 3.15.1 with default settings. Only contigs exceeding 500 nucleotides in length were considered for further analysis. Gene annotation was carried out on contigs using the Prokaryotic Genome-finding Algorithm, Prodigal [43].

A genomic comparative analysis was carried out to explore genomic variations between the adapted variants and the reference strain. The analysis of genetic mutation was conducted using Snippy (V4.6.0): https://github.com/tseemann/snippy, which uses bwa-mem for read mapping and Freebayes for variant calling. The following parameters were applied for SNP calling: a minimum quality score of 100 (*--minqual 100*), a minimum coverage of 10 (*--mincov 10*) and a minimum allele fraction of 0.9 (*--minfrac 0.9*) [44, 45]. In this study, *L. monocytogenes* ATCC 19,111 was used as the reference genome. The identified variants were evaluated across both coding and intergenic regions to capture potential mutations affecting genomic regions beyond protein-coding sequences. However, as this analysis was based on short-read sequencing and SNP-focused variant calling, large structural variations and copy-number variations were not specifically assessed.

### Statistical analysis

A statistical significance threshold of *p* < 0.05 was applied for the comparison MIC value fold change between groups, using a one-way ANOVA to assess overall differences between groups, followed by Tukey’s HSD post-hoc test for multiple pairwise comparisons, to ensure overall family-wise error rat control. Statistical assumptions of normality and homoscedasticity were verified prior to analysis. The bar plots illustrating bactericidal versus bacteriostatic activity were generated with GraphPad Prism version 10.

Metabolic activity values were calculated considering the average of the 10 highest absorbance values per metabolite among the wild type and the five variants. Relative activity [delta activity] was then calculated as follows: delta activity = activity (variant)/activity (wild type). Delta activities strictly less than 1 were transformed according to the following formula: Transformed delta activity = -(1/(delta activity). The average activity and relative activity rings were plotted using the Matplotlib and NumPy packages (v2.2.5) on a Python v3.10.6 kernel [46, 47]. The hierarchical clustering heat maps were constructed using R software (version 4.4.2), using distance correlation measures to build the dendrograms.

## Results

### Antimicrobial efficacy of antimicrobial peptides

In the current study, five antimicrobial peptides were selected based on their structural diversity and different mechanisms of action. The class IIa bacteriocin pediocin PA-1 (M31L) targets the bacterial mannose phosphotransferase system (Man-PTS), inducing pore formation and disrupting cell integrity. Nisin Z, a class Ia bacteriocin, primarily interacts with lipid II, an essential component of bacterial wall synthesis, providing a mechanism of action involving pore formation or blocking bacterial cell wall synthesis depending on the targeted bacterium. The class IIb bacteriocin plantaricin S, similarly, exerts a pore-forming effect on cell membranes by targeting cell wall phospholipids, leading to leakage of the intracellular components and the subsequent bacterial lethality. On the other hand, the cationic lipopeptide brevibacillin, which specifically targets bacterial lipoteichoic acid (LTA) and acts by disrupting their cytoplasmic membranes, was also selected for this study. These various mechanisms of action highlight the functional diversity of these peptides and their potential as effective antimicrobial agents against a wide range of pathogens, including *L.monocytogenes*.

All peptides were produced by chemical synthesis or salting out in good yields, and their purity above 90%, and identity confirmed by HPLC and mass spectrometry (Supplementary material Table S1 and Fig.S1). The antimicrobial activity of each peptide was evaluated qualitatively and quantitatively against a panel of *L.monocytogenes* isolates (*n* = 20) using microdilution assay through the determination of their MIC and MBC values using the Biolog system. The MIC and MBC values are presented in Table 1. Furthermore, the agar diffusion assay results, providing a visual assessment of antimicrobial activity, are shown in Fig.S2 of the supplementary material.

**Table 1:** Minimum inhibitory and bactericidal concentration values (µM) of the produced peptides against a panel of *L. monocytogenes* isolates

HPB	Serotype	Origin	PED		BN		BA		NIZ		PLS	
			MIC	MBC	MIC	MBC	MIC	MBC	MIC	MBC	MIC	MBC
2	1/2a	Food (coleslaw)	0.68	1.36	3.94	7.88	3.90	3.90	7.50	30.00	0.54	1.08
3	4b	Clinical	10.80	43.23	7.88	7.88	7.80	7.80	3.75	15.00	4.32	17.28
12	1/2a	Clinical (spinal fluid)	1.35	5.40	1.97	3.94	3.90	7.80	3.75	7.50	1.08	1.08
16	3a	Clinical	2.70	10.80	3.94	7.88	7.80	7.80	3.75	15.00	0.54	2.16
23	4d	Clinical	2.70	5.40	3.94	3.94	1.95	3.90	3.75	15.00	1.08	1.08
394	1/2b	Food (brie cheese)	1.35	10.80	1.97	1.97	1.95	3.90	15.00	30.00	1.08	2.16
399	1/2a	Clinical	0.68	1.35	1.97	1.97	1.95	1.95	3.75	30.00	1.08	8.64
412	1/2a	Clinical	5.40	43.23	1.97	1.97	3.90	3.90	3.75	30.00	1.08	2.16
413	1/2a	Clinical	0.68	1.36	1.97	1.97	3.90	3.90	7.50	15.00	0.54	8.64
568	1/2a	Food (cheese)	2.70	5.40	1.97	3.94	1.95	1.95	3.75	7.50	1.08	1.08
769	4b	Clinical (blood)	0.68	1.35	3.94	7.88	3.90	3.90	7.50	15.00	1.08	2.16
774	4c	Clinical	0.68	5.40	3.94	3.94	1.95	3.90	7.50	30.00	1.08	1.08
850	4b	Clinical	10.80	21.61	7.88	7.88	7.80	7.80	7.50	7.50	2.16	4.32
861	4b	Food (alfalfa tablets)	1.35	10.80	1.97	3.94	1.95	1.95	7.50	15.00	0.54	1.08
1088	4d	Clinical	1.35	10.80	1.97	1.97	3.90	3.90	7.50	60.00	0.54	1.08
1102	4b	Clinical	2.70	21.61	7.88	7.88	7.80	7.80	7.50	15.00	0.54	1.08
1340	1/2b	Clinical	10.80	21.61	7.88	7.88	7.80	7.80	3.75	7.50	2.16	4.32
1836	1/2a	Clinical	2.70	5.40	3.94	3.94	1.95	3.90	7.50	60.00	2.16	2.16
3034	4a	Clinical	1.35	5.40	3.94	7.88	1.95	1.95	7.50	30.00	0.54	4.32
5622	1/2a	Clinical (blood)	10.80	43.23	1.97	1.97	3.90	3.90	3.75	15.00	0.54	0.54

*Abbreviations*: PED, BN, BNA, NIZ and PLS are respectively, pediocin PA-1 (M31L), brevibacillin, brevibacillin Thr1,nisin Z and plantaricin S. HPB represent the Health Products Branch

**Fig. 2:**
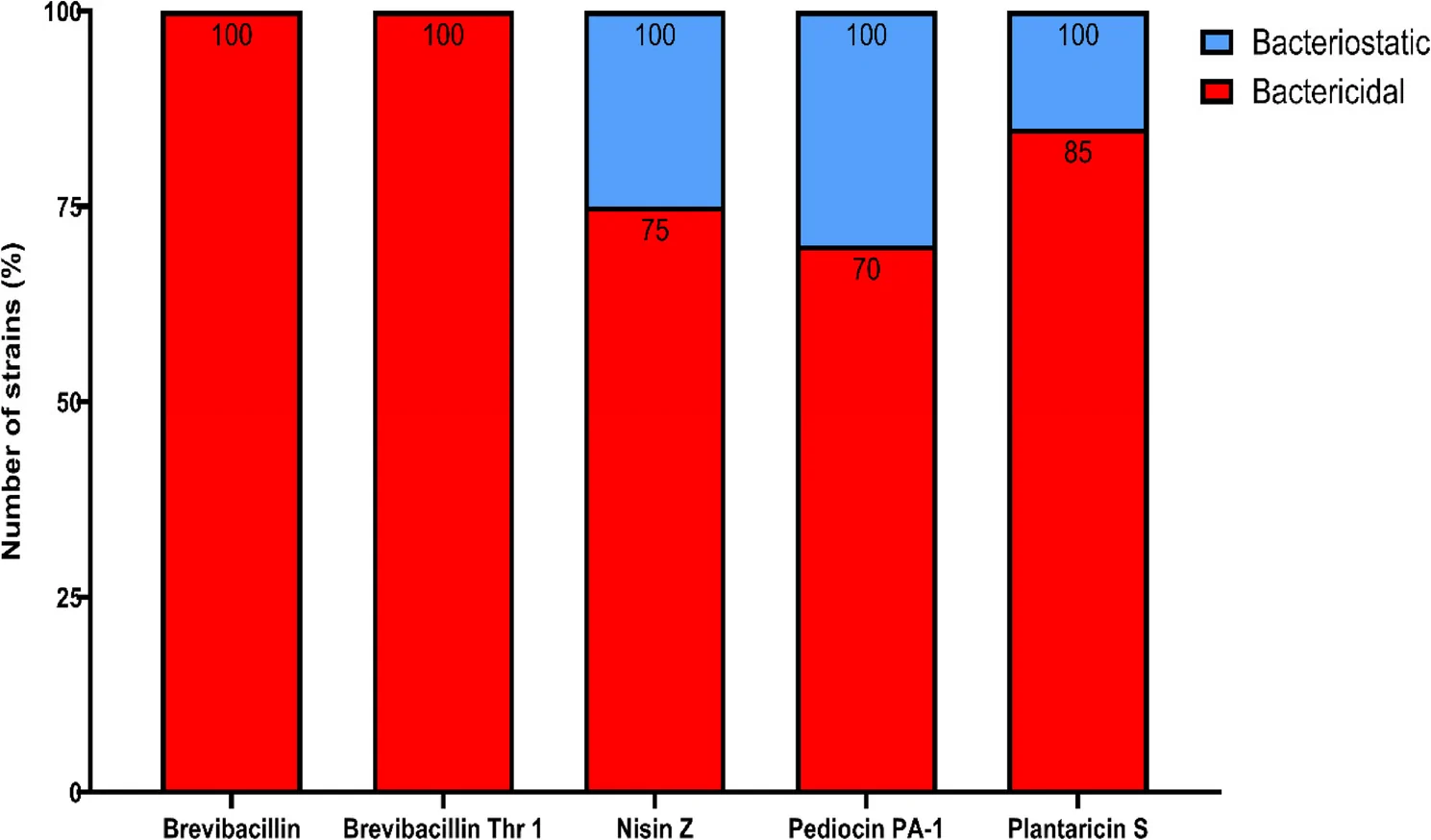
Antimicrobial effects of the different antimicrobial peptides against the panel of *L. monocytogenes* isolates (*n* = 20)

The results indicated that all *L.monocytogenes* isolates (*n* = 20) were susceptible to all tested peptides at different levels, with MIC values ranging from 0.54 to 15.00 µM (Table 1) and MBC values ranging from 1.08 to 60.00 µM (Table 1). Among the tested AMPs, plantaricin S, brevibacillin and its analog, were shown to be the most effective, exhibiting the lowest MIC and MBC values, compared to those obtained with pediocin PA-1 (M31L) and nisin Z. This variability in the inhibition activity of the tested peptides is probably linked to their distinct chemical structures and specific mechanisms of action. The calculated R ratios (Table S2) demonstrated that the antimicrobial activity of the peptides varies from bactericidal to bacteriostatic, depending on the tested molecules and the targeted bacterial strains, as illustrated in Fig. 2. More specifically, brevibacillin and its analog brevibacillinThr1 are characterized by a predominantly bactericidal activity. Meanwhile, plantaricin S, nisin Z, and pediocin PA-1 (M31L) have shown bactericidal activity against most of the tested strains, while occasionally exhibiting a bacteriostatic effect against specific isolates.

### Assessment of resistance, co- and cross-resistance between antimicrobial peptides

**Fig. 3:**
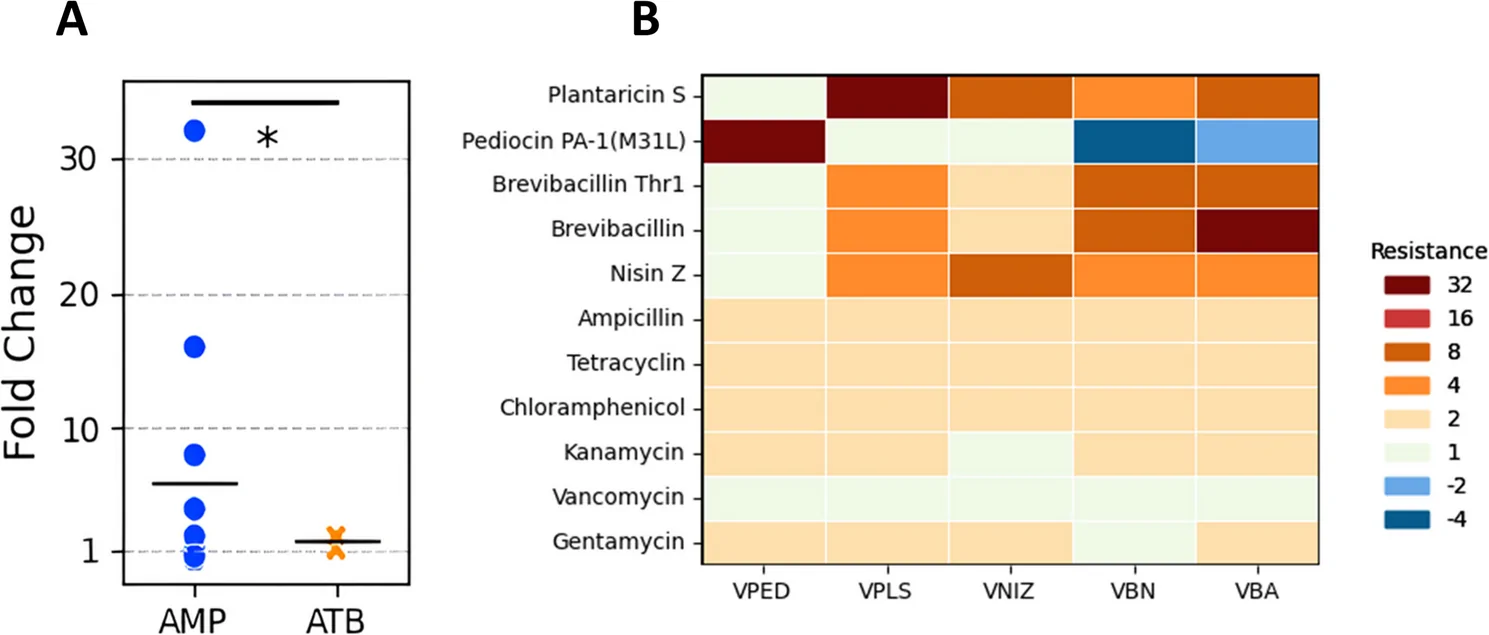
Change in MIC values for the tested AMPs and antibiotics (ATB) in AMP-adapted variants compared with the parental strain Listeria monocytogenes ATCC 19,111. **A** Fold-change in MIC values illustrating the overall susceptibility changes following AMP adaptation. **B** Heatmap representation showing the specific resistance and cross-resistance patterns of each AMP-adapted variant against the tested AMPs and antibiotics. *Represent a significant difference in the MIC’s values compared with the paternal strain (*p* < 0.05)

The susceptibility of different resistant variants to selected AMPs and antibiotics was assessed using agar diffusion assays and microdilution methods. These approaches were designed to identify co- and cross-resistance patterns between AMPs, as well as between AMPs and antibiotics. The wild-type *L.monocytogenes* ATCC 19,111 strain was shown to be sensitive to all the antimicrobial peptides, with the plantaricin S, nisin Z, pediocin PA-1, brevibacillin and its Thr1 analog, showing MIC values of 6.25, 25, 6.25, 6.25 and 3.125 µg.mL^− 1^, respectively. Meanwhile, the MIC values of 0.5, 0.125, 2, 2, 1 and 0.50 µg.mL^− 1^ were obtained for ampicillin, tetracycline, chloramphenicol, kanamycin, vancomycin and gentamicin, respectively (Table 2; Fig. 3).

**Table 2:** Minimal inhibitory concentration (µg.mL^-1^) of selected antimicrobial peptides and antibiotics against *L.monocytogenes* ATCC 19,111 and its AMP-resistant variants

L.monocytogenes variants						
Antimicrobial	WT*	vPED*	vPLS*	vNIZ*	vBN*	vBA*
Plantaricin S	6.25	6.25	100	50	25	50
Pediocin PA-1 (M31L)	6.25	200	6.25	6.25	1.56	3.125
Brevibacillin Thr1	6.25	6.25	25	12.5	50	50
Brevibacillin	3.125	3.125	12.5	6.25	25	50
Nisin Z	25	25	100	200	100	100
Ampicillin	0.5	1	1	1	1	1
Tetracycline	0.125	0.25	0.25	0.25	0.25	0.25
Chloramphenicol	2	4	4	4	4	4
Kanamycin	2	4	4	2	4	4
Vancomycin	1	1	1	1	1	1
Gentamicin	0.5	1	1	1	0.5	1

*Abbreviations*: WT, vPED, vPLS, vNIZ, vBN and vBA are respectively wild type strain, pediocin PA-1 (M31L), plantaricin S, nisin Z, brevibacillin and its Thr1 analog resistant variants were obtained by exposing *L.monocytogenes* ATCC 19,111 to increasing doses of each peptide from one quarter to ten times their MIC values. The MIC values were expressed in µg.ml^− 1^

*Adapted variants were generated independently in three separate experiments

Starting with the parent strain *L.monocytogenes* ATCC 19,111, the resistant variants were generated in a stepwise exposure to up 10X the MIC of a selected AMP. Afterward, cells resistant to pediocin PA-1 (vPED), to plantaricin S (vPLS), to nisin Z (vNIZ), to brevibacillin (vBN) and its Thr1 analog (vBA) were successfully isolated. The vPED adapted variants tolerated concentrations up to 200 µg.mL^− 1^ of pediocin PA-1 (M31L), corresponding to more than 30-fold increase in MIC compared to the wild-type strain (Fig. 3; Table 2). The vPED mutant did not show any cross-resistance to the other AMPs but a modest 2-fold decrease in sensitivity to ampicillin, tetracycline, chloramphenicol, kanamycin and gentamicin. This modest increase in MIC values against those antibiotics was mostly observed for all variants selected for resistance to the different AMPs. Importantly, none of the isolates demonstrated cross-resistance to vancomycin. Similarly, the plantaricin S-resistant mutant was not affected until the concentration of plantaricin S reached 100 µg.mL^− 1^, which is 30-fold higher than the initial MIC value obtained with the wild-type strain. Interestingly, resistance to plantaricin S confers a cross-resistance to nisin Z, brevibacillin and its Thr1 analog with MIC values 4-fold higher than those obtained with the parent strain. In addition, repeated exposure of *L.monocytogenes* ATCC 19,111 to high concentrations of nisin Z resulted in the emergence of a resistant mutant, able to tolerate concentrations of 200 µg.mL^− 1^, corresponding to a 16-fold increase in MIC value. 

Moreover, this acquired resistance to nisin Z also led to a significant increase in MIC values for plantaricin S (8-fold) as well as brevibacillin and its Thr1 analog (4-fold). Co-resistance was again observed between nisin Z and plantaricin S. However, non-significant or no change in terms of MIC value was observed for pediocin PA-1 (M31L) and other selected antibiotics. As observed with other AMPs, adapted variants generated with brevibacillin and its Thr1 analog yielded variants highly tolerant to both lipopeptides with an 8-fold increase in MIC values compared to the parent strain. The obtained results also showed that both variants were resistant to nisin Z and plantaricin S, indicating a cross-resistance between these AMPs. The only exception was pediocin PA-1, where the results showed a significant 2- to 4-fold reduction in MIC values from 6.25 µg.mL^− 1^ for the sensitive strain to 1.56 µg.mL^− 1^ and 3.125 µg.mL^− 1^ for the variants resistant to brevibacillin and its Thr1 analog, respectively. Most importantly, non-significant changes MIC values or no strong cross-resistance were observed with the antibiotics. These values were marginally increased by one-factor for ampicillin, tetracycline, chloramphenicol and kanamycin, while those for vancomycin remained unchanged.

### Agar diffusion assay

**Fig. 4:**
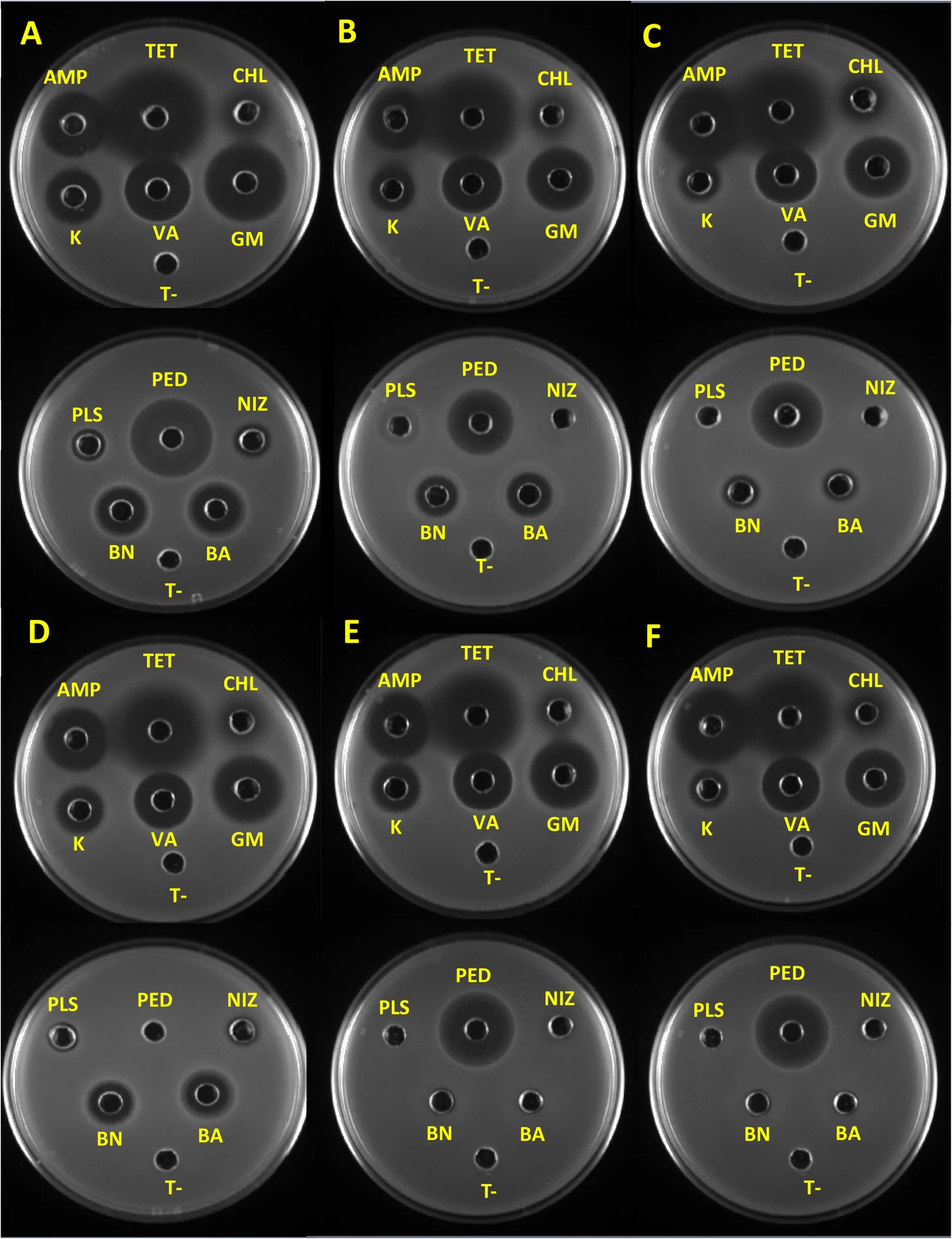
Agar diffusion test showing cross-resistance between AMPs and antibiotics. The agar was inoculated with *L. monocytogenes* ATCC 19,111 (**A**) and its resistant variants vNIZ (**B**), VPLS (**C**), vPED (**D**), vBN (**E**) and vBA (**F**). The wells respectively contain 80 µL of different AMPs at a concentration of 200 µg.mL-1 and 10 µg.mL-1 for the antibiotics. AMP, CHL, GM, K, TET, and VA are respectively ampicillin, chloramphenicol, gentamicin, kanamycin, tetracycline and vancomycin. Sterile ultrapure water with 10% DMSO were used as a negative control (T-)

The qualitative analysis of sensitivity of vPED, vBA, vBN, vPLS and vNIZ variants to different AMPs using an agar diffusion assay revealed different susceptibility profiles, highlighting the observed co and cross-resistance pattern. The different inhibition zones and diameters are shown in Fig. 4; Table 3.

**Table 3:** Antimicrobial susceptibility of *L. monocytogenes* ATCC 19,111 and its AMP-resistant variants

Inhibition zone diameters (mm)						
Peptides/Antibiotics	WT	vPED	vPLS	vNIZ	vBN	vBA
Plantaricin S	11.00 ± 0.10	10.06 ± 0.05	-	-	-	-
Pediocin PA-1 (M31L)	27.23 ± 0.58	-	25.00 ± 0.10	23.96 ± 0.05	25.06 ± 0.05	24.10 ± 0.10
Brevibacillin Thr1	20.06 ± 0.05	19.50 ± 0.10	16.50 ± 0.10	18.40 ± 0.10	-	-
Brevibacillin	19.53 ± 0.05	18.43 ± 0.05	17.06 ± 0.05	18.00 ± 0.10	-	-
Nisin Z	10.90 ± 0.10	10.00 ± 0.10	-	-	-	-
Ampicillin	20.90 ± 0.10	19.96 ± 0.05	21.90 ± 0.10	21.10 ± 0.10	19.90 ± 0.10	20.96 ± 0.05
Tetracycline	35.90 ± 0.10	35.06 ± 0.05	36.10 ± 0.10	35.00 ± 0.10	35.10 ± 0.10	35.00 ± 0.10
Chloramphenicol	17.00 ± 0.10	15.90 ± 0.10	16.00 ± 0.10	16.00 ± 0.10	16.00 ± 0.10	17.00 ± 0.10
Kanamycin	16.00 ± 0.10	15.10 ± 0.10	14.93 ± 0.05	16.06 ± 0.05	16.10 ± 0.10	16.06 ± 0.10
Vancomycin	19.00 ± 0.10	19.06 ± 0.05	19.06 ± 0.05	18.93 ± 0.05	18.90 ± 0.10	18.10 ± 0.10
Gentamicin	30.10 ± 0.10	28.03 ± 0.05	29.03 ± 0.11	29.06 ± 0.11	29.03 ± 0.15	28.00 ± 0.10

*Abbreviations*: (-) indicate that no inhibition zone was identified

Our data revealed that all adapted variants, except for vPED, maintained significant susceptibility to pediocin PA-1(M31L), exhibiting an inhibition zone averaging between 23.96 ± 0.05 mm and 25.00 ± 0.10 mm, relatively close to those observed for the wild-type strain (WT, 27.23 ± 0.58 mm). The vBA displayed no visible inhibition zones towards nisin Z, plantaricin S and brevibacillin and a modest decrease in susceptibility was observed for pediocin PA-1 (M31L), with an inhibition zone of 24.10 ± 0.10 mm (Fig. 4). A similar pattern was observed in vBN, vPLS and vNIZ, demonstrating co-resistance and cross-resistance between these different antimicrobial agents (Fig. 4). In addition, both vPLS and vNIZ variants share similar susceptibility profiles to the different AMPs, with an apparent co-resistance between plantaricin S and nisin Z and reduced susceptibility to brevibacillin and its Thr1 analog, as indicated by the inhibition zones. Overall, all adapted variants remained susceptible to all antibiotics. Indeed, variants resistant to plantaricin S (vPLS), nisin Z (vNIZ), native brevibacillin (vBN) and its Thr1 analog (vBA) demonstrated a high susceptibility to the different antibiotics similar to that obtained with WT strain. Taken together, these results, clearly demonstrate the absence of cross-resistance between the selected AMPs and conventional antibiotics.

### AMPs-resistant variants specific growth rates

**Fig. 5:**
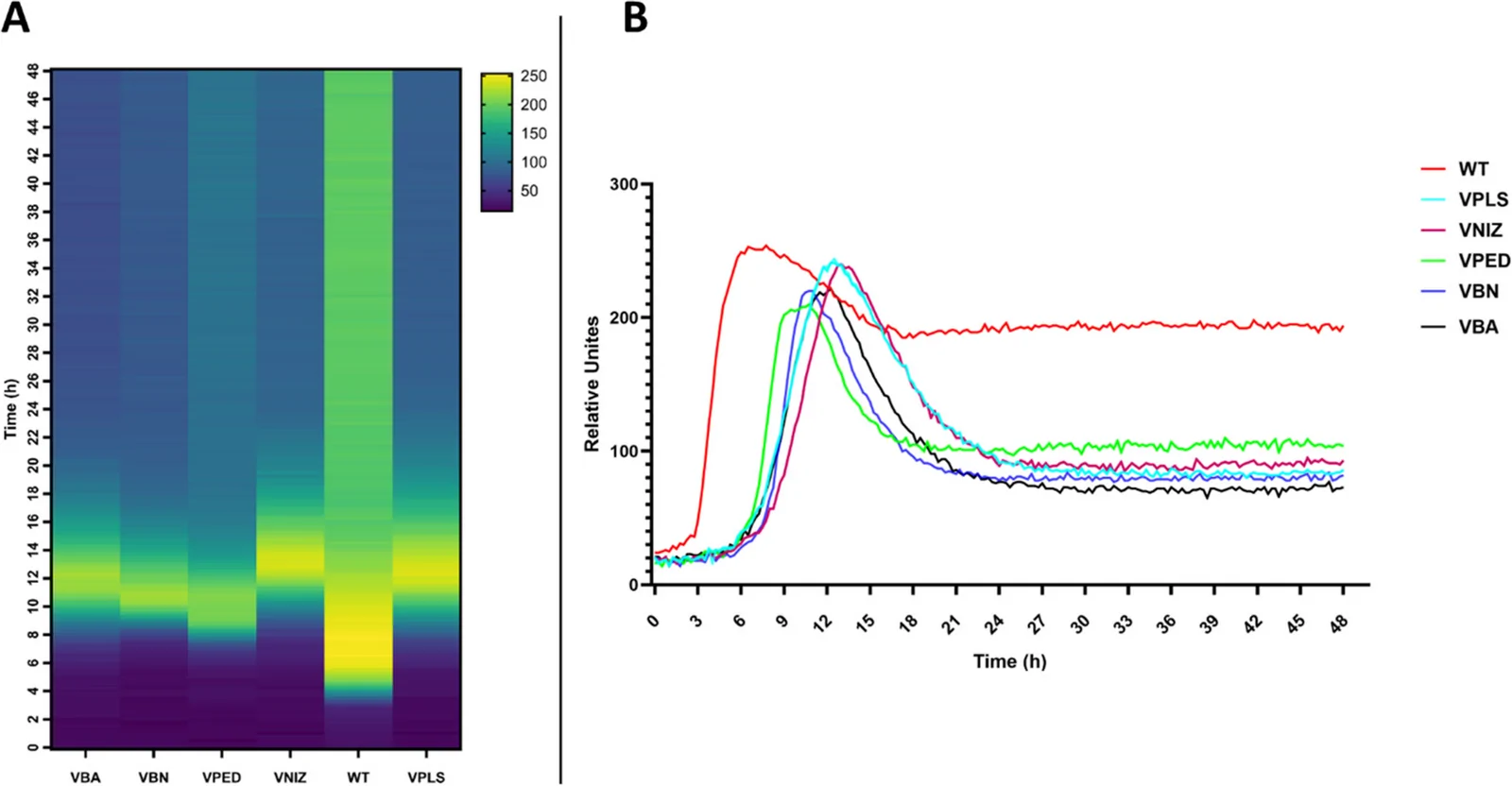
The specific growth rate of *Listeria monocytogenes* ATCC 19,111 (WT) and its resistant variants. **A** Heatmap representing the expression intensity of the growth signal among different AMP-resistant variants over time. Higher growth intensity is indicated by a more yellow color. **B** Graphical representation of the growth curve for each AMP-mutant, allowing comparison of induction, maximum intensity, and stabilization of the growth signal. Growth rates are expressed in relative units (RU) for each mutant

The specific growth rate of the reference strain *L. monocytogenes* ATCC 19,111 (WT) and the different resistant variants is shown in Fig. 5. In general, our data revealed a lower growth rate among all variants compared to the WT strain. The vPED mutant exhibited the lowest growth rates with 35% reduction compared to the WT strain. This may indicate a high biological cost associated with the mutation conferring this resistance. While the vPLS demonstrated 25% less growth, the nisin-resistant variant vNIZ showed an average of 20% less growth. Finally, brevibacillin-resistant variants vBN and vBA appeared to be less affected with only 22% and 18% less growth, respectively. When the adapted variants growth kinetics are compared across AMPs-slected variants, vPED clearly emerged as the most impacted with a significant average growth deviation compared to all other variants (approximately 10–17% additional growth reduction). Despite being associated with the same antimicrobial class, vPLS and vNIZ showed a slightly similar growth pattern with only 5% difference compared to the respective adapted variants. Reflecting a relatively comparable impact on cell dynamics. Moreover, vBN and vBA variants display considerable variation, with vBA being around an additional 4% less affected compared to vBN, potentially reflecting specific differences in resistance mechanisms or better adaptation of vBA.

### Comparison of fitness cost on the AMPs resistant variants using high-throughput phenotyping assay

**Fig. 6:**
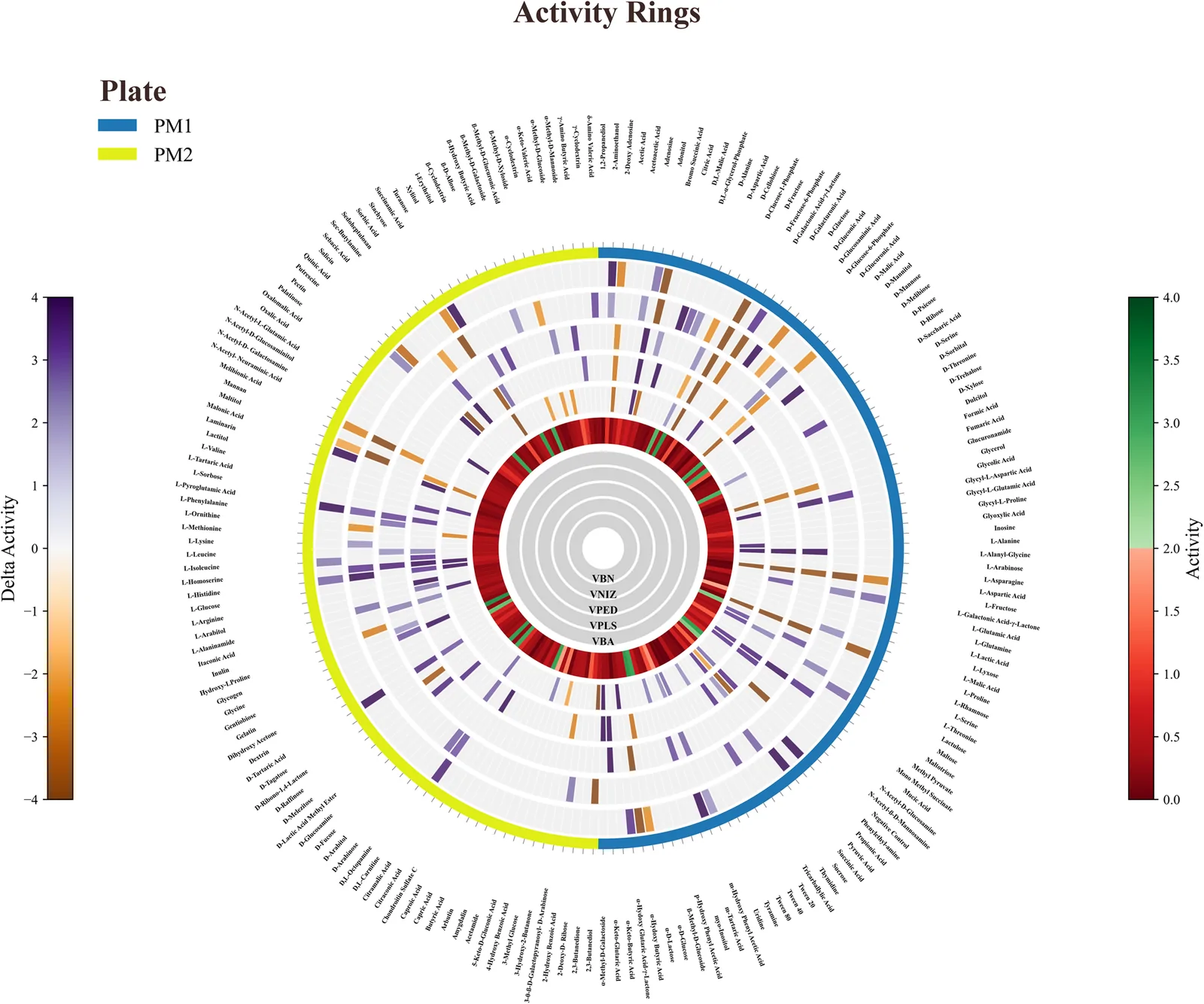
Overall growth/metabolic activity ring based and phenotype microarray comparison of the strains to carbon and nitrogen sources utilization (PM1 and PM2). Internal grey circle represents the resistant variant order; the external blue and yellow circles indicate the PM categories. The activity index (AI) calculated using each strain and each well was shown as a colour band ranging from red (AI = 0) to green (AI = 4). Delta activity is represented as the difference from the AI value corresponding to the reference strain being equal to or higher than 2 AI; grey indicates no difference; purple indicates higher activity; orange indicates lower activity

**Fig. 7:**
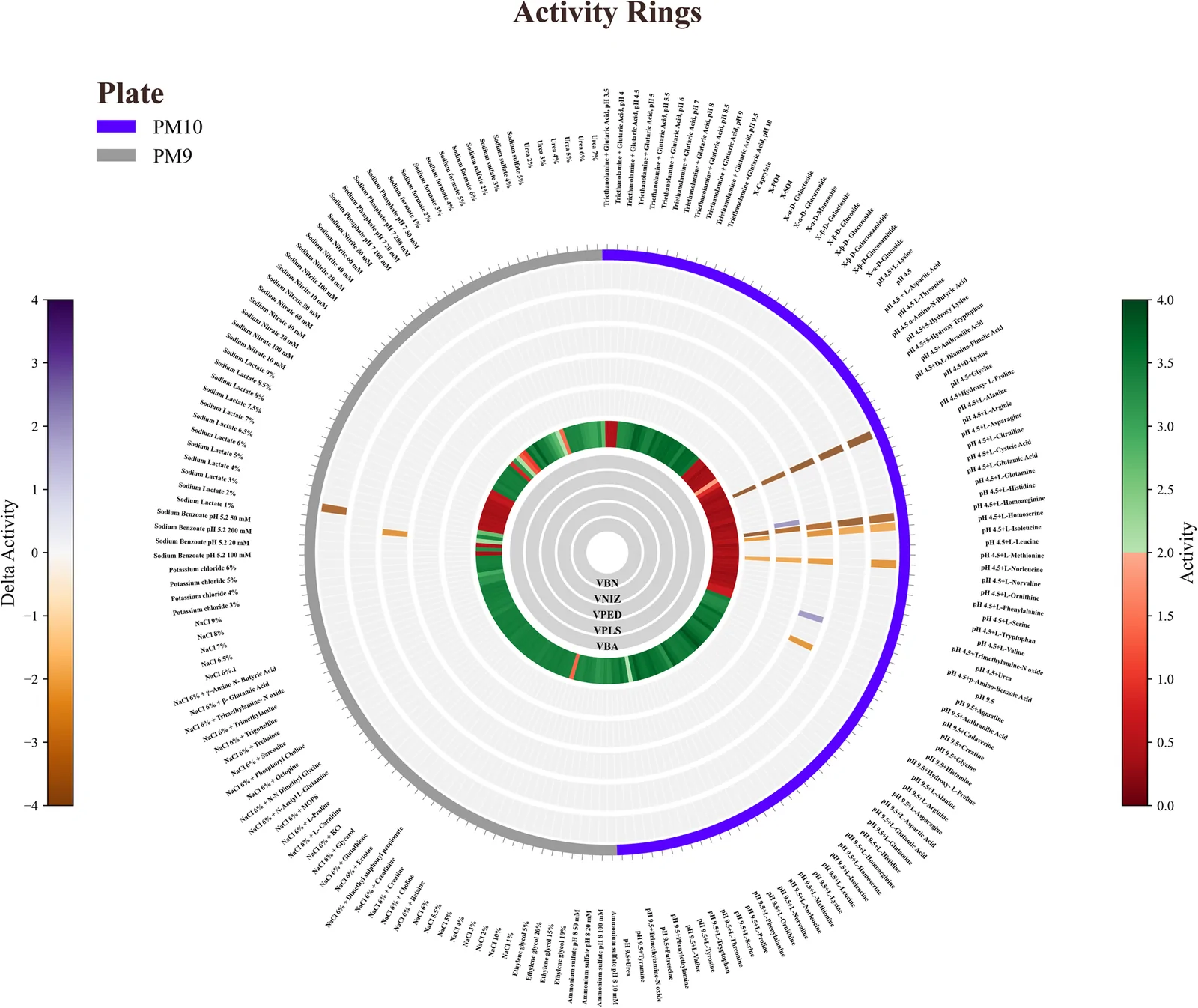
Overall growth/metabolic activity ring based and phenotype microarray comparison of the strains to osmolytes and pH stress adaptation (PM9 and PM10). Internal grey circle represents the AMP-resistant variant order; the external purple and grey circle indicate the PM categories. The activity index (AI) calculated using each strain and each well was shown as a colour band ranging from red (AI = 0) to green (AI = 4). Delta activity is represented as the difference from the AI value corresponding to the reference strain being equal to or higher than 2 AI; grey indicates no difference; purple indicates higher activity; orange indicates lower activity

The metabolic profile analysis across the five AMP-resistant variants has revealed a substantially higher relative consumption of several substrates compared to the wild-type strain. The relative substrate consumption was quantified using the mean of the ten most representative absorbance values for each variant and was expressed as a percentage by comparison to the wild-type strain. The variants and substrates were grouped into activity rings and distinct clusters by Euclidean distance analysis as shown in Figs. 6 and 7 as well as Fig.S3 to S6 in the supplementary material. 

Quantitative assessment of carbon and nitrogen sources utilization has shown that both vBA and vPED exhibit increased substrate-dependant metabolic activity on several substrates involved in the glycolytic and energetic pathways, namely D-fructose, D-glucose-6-phosphate, pyruvate and citrate, suggesting altered utilization of simple sugars and central metabolic intermediates of the tricarboxylic acid cycle (TCA) cycle [29]. Moreover, these variants were characterized by a high uptake of organic acids such as butyric, malonic and oxalomalic acid, suggesting a potential shift in the utilization of alternative carbon sources related to TCA cycle intermediates [48]. On the other hand, a significant reduction in the absorption of amino acids such as L-serine, L-asparagine and L-threonine was observed, indicating differences in amino acid substrate utilization that may reflect changes in associated metabolic pathways [28]. Furthermore, a decreased consumption of D-arabinose, D-raffinose and amygdalin was noted, suggesting a reduced capacity for the utilization of certain complex sugars and glycosides [49, 50].

The nisin-resistant variant (vNIZ) showed a distinctive metabolic profile, marked by high consumption of α-hydroxyglutaric acid-γ-lactone and myo-inositol, two substrates involved in sugar secondary pathways and polyol metabolism [51, 52]. In addition, an increased phenotypic microarray responses on high-nitrogen substrates such as L-arginine, L-histidine and putrescine was observed, suggesting differences in nitrogen-containing substrate utilization and potential alterations in polyamine-related metabolism [53–55]. Meanwhile, vNIZ has a limited consumption of key energy substrates such as D-mannitol, D-sorbitol and maltose, which could indicate deficient transport systems or inhibition of certain enzymes associated with the metabolism of complex sugars [56–59]. In addition, a low consumption of inulin, mannan and β-cyclodextrin was identified among this variant, suggesting a reduced capacity to metabolize certain carbohydrate polymers [60, 61].

Our data also show that the plantaricin S-variant (vPLS) is characterized by an increased consumption of organic acids such as succinic acid, propionic acid and malic acid, suggesting differential utilization of organic acid substrates associated with central metabolism [62–64]. Furthermore, this mutant showed preferential uptake of polyols and carbohydrate derivatives, particularly xylitol, arbutin and D-arabitol, indicating a distinct substrate utilization profile involving secondary sugar metabolism pathways [65, 66]. In contrast, the vPLS mutant showed a reduced consumption of nitrogen-rich substrates such as glycine, adenosine and uridine, which may reflect differentiated regulation of nucleotide and amino acid metabolism [67, 68]. Moreover, its low uptake of hydroxy-L-proline, glycine and L-methionine suggests an altered metabolism of sulfurated amino acids and hydroxyl derivatives [69–71]. Finally, the brevibacillin-resistant variant (vBN) is marked by high consumption of acetic, D-galacturonic and D-gluconic acids, indicating an increased capacity to utilize specific carbohydrate-derived acids [72]. High metabolism of carboxylates and complex sugars was also observed, with enhanced metabolic phenotype on citramalic acid, 5-keto-D-gluconic acid and sorbic acid, indicating efficient utilization of oxidized carbohydrate-derived substrates [73, 74]. By contrast, vBA variant has displayed markedly decreased consumption of L-proline, inosine and D-ribose, suggesting altered utilization of substrates associated with nucleotide metabolism and amino acid catabolism [70]. Moreover, lower consumption of L-leucine, L-isoleucine and γ-aminobutyric acid (GABA) was observed for this mutant, which could indicate a reduced capacity to metabolize certain branched-chain amino acids [75, 76].

Our comparative analysis of the osmotic and ionic stress tolerance profiles of the five AMP-resistant variants have revealed significant variations in their ability to tolerate salts, organic acids and several nitrogen sources. The vPED and vBA variants exhibit an increased tolerance to high NaCl concentrations (up to 10%), particularly in the presence of osmoprotectants such as L-proline, glycine betaine, creatine and trimethylamine-N-oxide, indicating an enhanced regulation of osmoregulatory mechanisms [77, 78].

These variants have also shown an increased resistance to nitrates and nitrites (up to 100 mM NaNO₃ and NaNO₂) as well as sodium benzoate (up to 200 mM, pH 5.2), indicating an improved ability to cope with oxidative stress and the toxicity of the antimicrobial agents [79, 80]. However, vBN and vNIZ variants demonstrated an amplified sensitivity to sodium benzoate (≥ 100 mM), sodium sulphate (≥ 4%) and sodium phosphate (≥ 200 mM, pH 7), potentially reflecting an alteration of the ionic transport systems and detoxification mechanisms [81]. Furthermore, vPLS showed a preferential tolerance to saline solutions especially when combined with osmoprotectants such as ectoine, trehalose and sarcosine, which suggests an increased reliance on external solutions to maintain osmotic homeostasis [82]. In addition, vBA and vPED were more efficient at metabolizing sodium lactate (up to 9%), sodium formate (up to 6%) and urea (up to 7%), compared with vBN and vNIZ, which showed a reduced metabolic phenotype on these substrates, indicating a differential pattern of energy exploitation under stress conditions. The clustering and Euclidean distance analysis allowed us to group the variants according to their ability to adapt to ionic and chemical stresses. This approach revealed that vPED and vBA emphasize a strategy of active resistance to salts and organic acids, while vBN and vNIZ appear to be more sensitive to oxidative and ionic stresses, and vPLS mainly adapts via the uptake of exogenous osmoprotectants. These results highlight distinctive stress tolerance strategies among the studied variants, underscoring the impact of mutations on ionic and metabolic regulatory mechanisms.

The comparative analysis of AMP-resistant variants revealed distinct differences in their response to pH variations and the uses of organic substrates. The vPED and vNIZ were characterized by higher metabolic activity under acidic assay conditions (pH 4.5), particularly in the presence of 5-hydroxy tryptophan, L-histidine and anthranilic acid, suggesting differences in substrate utilization under acidic conditions [83]. By contrast, vBN and vPLS have a marked distinct metabolic profile under alkaline assay conditions (pH 9.5), with significant utilization of L-tryptophan, L-arginine, L-ornithine and L-phenylalanine, indicating differences in nitrogen-containing substrate utilization under basic conditions [84]. In the case of VBA, a broader substrate utilization profile was adopted at pH 4.5 and 9.5, with a preference for L-homoserine, L-phenylalanine and L-methionine, suggesting greater metabolic versatility. Additionally, the growth profiles have revealed that vPED and vNIZ have responded differently to certain amino acids such as L-lysine and L-homoarginine, while vBN and vPLS have used more effectively, L-valine, L-isoleucine and L-tyrosine.

### Genetic determinants underlying AMPs resistance acquisition

A comparative genomic study was also conducted to identify genetic determinants potentially associated with the acquired resistance phenotypes. The identification of stable genetic variations in the AMP-adapted variants provided evidence that the increased resistance observed following serial peptide exposure was associated with heritable genomic changes rather than solely resulting from transient physiological adaptation. A comparative analysis was carried out on single-nucleotide polymorphism (SNP) profiles, focusing on non-synonymous variation SNPs significantly detected in coding sequences (CDs). Genomic analysis revealed between 1 and 4 SNPs for each AMP-resistant variant. These genomic alterations were maintained in the selected lineages used for downstream phenotypic analyses, supporting the stability of the acquired resistance phenotype. The SNPs associated with the different resistance profiles detected for each AMP are summarized in Table 4. The genome of the *L. monocytogenes* ATCC 19,111 was used as the reference strain.

**Table 4:** List of the detected genes mutations using the comparative pan-genomic analysis

Genes	mutations	Function	vPED	vPLS	vNIZ	vBA	vBN
*dgaR*	ATCC_19111_01004_p.Met234Ile	Transcriptional regulatory protein DagR	**+** (*n* = 15)	**-**	**-**	**-**	**-**
*yofA*	ATCC_19111_01498_p.Gly273_Lys274insertionAsn_Gly	HTH-type transcriptional regulator YofA	**+** (*n* = 15)	**-**	**-**	**-**	**-**
*PrfA*	ATCC_19111_02954_p.Gly44Cys	Listeriolysin regulatory protein	**+** (*n* = 15)	**-**	**-**	**-**	**-**
*walK*	ATCC_19111_01512_p.Met26Ile	Sensor histidine kinase WalK	**-**	**-**	**+** (*n* = 11)	**-**	**-**
*walR*	ATCC_19111_01514_p.Arg67Cys	Transcriptional regulatory protein WalR	**-**	**-**	**+** (*n* = 11)	**-**	**-**
*crnA*	ATCC_19111_02240_p.Ser216Ile	Creatinine amidohydrolase	**-**	**-**	**-**	**+** (*n* = 11)	**+** (*n* = 15)
*topA*	ATCC_19111_00435_p.Asp646_Gln680delinsGlu	DNA topoisomerase	**-**	**-**	**-**	**+** (*n* = 11)	**+** (*n* = 15)
*plsX*	ATCC_19111_02073_p.Ala145Glu	Glutamine-fructose-6-phosphate aminotransferase	**-**	**-**	**-**	**+** (*n* = 11)	**+** (*n* = 15)
*menH*	ATCC_19111_02577_p.Gln29Lys	2-succinyl-6-hydroxy-2,4-cyclohexadiene-1-carboxylate synthase	**-**	**-**	**-**	**+** (*n* = 11)	**+** (*n* = 15)
*graR*	ATCC_19111_02863_p.Thr16Lys	Response regulator protein GraR	**-**	**-**	**+** (*n* = 11)	**-**	**-**
*degU*	ATCC_19111_02514_p.Ser181Ile	Transcriptional regulatory protein DegU	**-**	**+** (*n* = 15)	**-**	**-**	**-**

*Abbreviations*: (+) indicate that the mutation were detected for the selected gene and the (-) indicate that no mutation were detected. AMPs-adapted variants were generated independently in three separate experiments. (n): indicate the number of resistant colonies carrying the corresponding mutation

For the vPED, three variants were identified. The first observed mutated SNP was a Met234Ile mutation located in the transcriptional regulator gene, *dgaR*. The second involves a transcriptional regulator, *yofA* gene, mutated with an indel, involving the insertion of an asparagine and a glycine between positions 273 and 274 of the gene product [85]. This insertion could potentially alter YofA regulator ability to interact with DNA, thereby disrupting the expression of the genes and its regulation [85]. The Gly44Cys mutation in *prfA* gene, a transcriptional regulator factor of the virulence cascade in *L.monocytogenes* ATCC 19,111 [86].

For the nisin-adapted variant, three SNPs were also identified. The first mutated SNP (Met26Ile) was observed in the *walK* gene, encoding a sensory histidine kinase, known to be involved in regulating cell wall integrity and in the response to environmental stresses [87]. A second mutation was identified in the same operon, the transcriptional regulator WalR (Arg67Cys) [88]. The third mutation (Thr16Lys) was identified in the *graR* gene, encoding a response regulator protein (GraR) involved in resistance to daptomycin and cationic antimicrobial peptides [89]. For the plantaricin S-resistant variant, a single mutated SNP (Ile181Ser) was observed in the transcriptional regulator *degU* gene [90]. This mutation could potentially affect the expression of several virulence factors including motility and biofilm formation [90].

As some expected, the same four specific mutations were identified in both vBN and vBA. One SNP was in the *crnA* gene (Ser216), encoding a creatinine amidohydrolase involved in the nitrogen derivatives metabolism [91]. This mutation could potentially affect the enzymatic activity, thereby influencing metabolite regulation related to antimicrobial response. A second mutation (Gln29Lys) was in the *menH* gene, whose product is involved in the menaquinone (vitamin K2) biosynthesis [92]. This mutation might disrupt menaquinone production, compromising resistance mechanisms by affecting energetic processes and bacterial growth. The third observed mutation in vBN and vBA was in the *plsX* gene (Ala145Glu), coding for glutamine-fructose-6-phosphate aminotransferase, a key enzyme in phospholipid and fatty acid biosynthesis in Gram-positive bacteria [93]. The last mutation was found in the *topA* gene, where a single indel mutation was located in this gene (Table 4). A mutation in this gene, which codes for DNA topoisomerase, may impact bacterial stress response mechanisms and resistance to acidic conditions, such as the RpoS-dependent and glutamate decarboxylase (GAD) systems [94].

## Discussion

Antimicrobial peptides of bacterial origin have attracted increasing interest due to their potent antimicrobial properties and their potential applications in the food, medical and veterinary sectors, as well as their potential role as alternatives to conventional antimicrobials [95]. However, the lack of knowledge about the emergence and mechanisms of resistance to AMPs remains a major concern that hinders their large-scale implementation [96, 97]. Moreover, the extent of cross-resistance between different AMPs, as well as between AMPs and antibiotics, remains a field of research that needs to be investigated in order to provide a more rational overview regarding an effective management and exploitation of the AMPs.

In this study, we primarily evaluated the anti-*Listerial* activity of bacterial AMPs belonging to different classes, including three bacteriocins as well as two lipopeptides, where the antimicrobial activity is mainly related to interactions with the cell membranes of the targeted bacteria [31, 32, 98]. These AMPs were screened for their inhibitory activity against 20 *L.monocytogenes* isolates, from food and clinical sources. Our data revealed variable activity across the different isolates, as reflected by MIC values ranging from 0.54 µM to 15.00 µM and MBC values between 1.08 µM and 60.00 µM (Table 1). Additionally, the effectiveness of the tested AMPs showed variable effects, ranging from bacteriostatic to bactericidal. The obtained results showed that the observed effects were generally linked to a total bacterial eradication rather than growth inhibition, indicating a bactericidal effect as the main action of these peptides (Fig. 2).

Despite the high inhibitory potential of these molecules, their application remains limited due to the lack of detailed data regarding the development of resistance as well as the risk of cross-resistance between these AMPs and conventional antibiotics [99]. In this context, the evolutionary dynamics of the acquired resistance to these peptides were analyzed, using *L.monocytogenes* ATCC 19,111 as a model strain to generate resistant variants to nisin Z, pediocin PA-1, plantaricin S, brevibacillin and its Thr1 analogue. A previous study highlighted that a variant resistant to divergicin V41, a class IIa bacteriocin produced by Gram-positive bacteria, also exhibited co-resistance to piscicocins V1 a bacteriocin from the same class, while maintaining sensitivity to nisin [100]. These results are in line with those of Schillinger et al. 1998, who reported that *L.monocytogenes* Scott A nisin-resistant mutants maintained their susceptibility to other class IIa bacteriocins, namely sakacin A and enterocin B [101]. This pattern of resistance was also identified in our study, where the pediocin PA-1 resistant variant maintained sensitivity to nisin Z, as shown by the MIC values (Table 2) and zones of inhibition in the agar diffusion assay (Table 3; Fig. 4). Furthermore, co and cross-resistance was observed between vPLS and vNIZ, which show reciprocal resistance to nisin Z and plantaricin S, as well as brevibacillin-resistant variants (vBA and vBN), which are also resistant to these bacteriocins. These results raised important questions about the resistance mechanisms involved in these phenotypes.

In general no major cross-resistance has been observed between the tested AMPs and conventional antibiotics, which may be explained by the difference in mechanisms of action of these two classes of antimicrobials as previously reported [102]. Antibiotics, such as beta-lactams or aminoglycosides, are characterized by a variety of mechanisms of action, including inhibition of protein or DNA synthesis, or disruption of cell wall function mediated by targets that the ones targeted by bacteriocins and lipopeptides [103, 104]. This difference in modes of action potentially mitigates the risk of cross-resistance between these two classes of antimicrobial agents, as also demonstrated by several studies [102]. Despite the fact that these AMPs display variable efficacy, they nevertheless have different mechanisms of actions, which could explain the emergence of cross-resistance that could be attributed to common membrane targets, while the involvement of genetic mutations and specific regulations in this phenomenon requires further investigation.

In order to investigate the mechanisms underlying the acquired resistance, co- and cross-resistance, a genomic analysis was carried out across numerous generations of bacterial resistant variants, progressively selected under increasing pressure conditions. We aimed to investigate the relationship between AMPs susceptibility profiles and the occurrence of specific mutations. The identification of these mutations enables a more in-depth assessment of the adaptive mechanisms involved in the development of resistance patterns. Our genomic analysis allowed the detection of specific gene mutations, which were considered potential genetic contributors associated with the AMP-resistant phenotypes observed in this study. Although these mutations provide biologically plausible explanations based on previous functional studies, their direct causal contribution to resistance requires further experimental validation. In particular, three SNPs have been specifically identified in vPED variants that are associated with pediocin PA-1 resistance. Mutations affecting the *dgaR*, *yofA* and *prfA* genes potentially promote resistance by modulating the gene expression involved in membrane stress response, regulation of cell wall synthesis and bacterial virulence [105]. As a transcriptional regulator, the DagR protein might be implicated in the repression or the activation of several metabolic pathways involved in the biosynthesis of bacterial cell wall components such as lipids or teichoic acids [106, 107]. Consequently, any mutation in this gene may contribute to cell-surface modifications that could affect pediocin PA-1 affinity toward its specific receptor, mainly the Man-PTS receptor, as demonstrated in *L.monocytogenes* [108]. Similarly, YofA, as an HTH regulator, could also influence the regulation of membrane integrity gene expression and ultimately leads to either changes in membrane fluidity or charge alterations, which may affect the AMPs ability to successfully integrate their active domains, thereby highlighting the interconnection between transcriptional regulation and cell membrane dynamics [85]. The *prfA* gene, known as a virulence regulator in *L.monocytogenes*, also regulates a wide range of cellular functions, including genes associated with membrane stress and metabolic response [86]. Mutations in *prfA* may contribute to increased tolerance to membrane perturbations, such as that induced by pediocin PA-1. This hypothesis is in line with observations showing that prfA-mutants may exhibit an altered membrane permeability or surface charge, affecting the effectiveness of cationic antimicrobial peptides [109]. Furthermore, recent transcriptomic studies performed in *L. monocytogenes* under bacteriocin exposure conditions have demonstrated that bacteriocin-induced stress can modulate the expression of virulence and adaptation-associated regulatory networks, including *prfA*, providing complementary evidence supporting the potential involvement of this regulator in the response to AMPs [110]. However, the mutation identified in *prfA* in the present study should be considered as a potential contributor associated with the observed phenotype rather than a directly validated determinant of pediocin PA-1 resistance.

For plantaricin S-resistant variants, a mutation has been identified in the *degU* gene, encoding the DegU regulatory protein which belongs to *Bacillus* spp two-component system (DegS-DegU), which play a key role in the environmental adaptation process [111, 112]. This mutation could be associated with increased tolerance to plantaricin S through potential alterations in the expression of several genes involved in cell wall structure and biofilm development. For example, some studies have demonstrated that *degU* gene mutations can induce an over-activation of extracellular proteases and an overproduction of exopolysaccharides, thereby potentially preventing the interaction of the AMP with its membrane target [113]. In agreement with these observations, recent studies investigating bacteriocin responses in *L. monocytogenes* have reported transcriptional modulation of regulatory pathways associated with adaptation, virulence and stress responses, including *degU*-related regulatory networks under bacteriocin exposure conditions in food-relevant environments [110, 114]. In addition, other studies have reported that certain *degU* mutations can induce cell membrane architectural alterations, leading to an alteration of surface charge or perturbation of the membrane fluidity, which may contribute to reduced interaction with cationic peptides such as plantaricin S [115]. These findings provide complementary support for the potential involvement of regulatory pathways in bacteriocin adaptation. However, the specific contribution of the identified *degU* mutation to plantaricin S resistance requires further functional validation.

Mutations in the *walK*, *walR*, and *graR* genes have been identified as potential genetic factors associated with the acquisition of resistance to nisin Z. Several studies have demonstrated that mutations in these genes lead to increased regulation of response systems, such as the GraRS two-component system, which is known for its involvement in the response to cationic AMPs, and may influence the regulation of defense-related gene expression, including the *vraFG* and *mprF* defence genes [116, 117]. The mprF defense factor act by modulating the cell membrane charge through the addition of lysine residues to phosphatidylglycerol, consequently reducing the electrostatic affinity of nisin Z towards the cell surface [117]. However, the VraFG transporter was shown to be involved in membrane homeostasis and cell surface property modulation, through membrane scavenging and the export of components modifying cell surface properties, which may contribute to reduced membrane accessibility to nisin [118, 119]. Furthermore, the interactive nature of the GraRS and walKR systems has been established, where mutations in *walK* and *walR* may contribute to resistance-associated phenotypes by modulating membrane-related signalling pathways [120, 121]. The WalKR system is essential for cellular viability, by regulating key genes implicated in peptidoglycan synthesis, cell division and autolysis [122]. Mutations in *walK* or *walR* genes can impair the activity of the system, potentially resulting in down-regulation of autolysis and an increase in cell wall thickness, subsequently limiting the penetration and antimicrobial efficacy of nisin Z [122].

The development of resistant variants to brevibacillin and its Thr1 analog could be related to adaptive mutations occurring in metabolism-related genes, including *menH*, *plsX*, *crnA* and *topA*. Although these genes do not directly encode classical resistance mechanisms, their alterations may modify bacterial physiology and potentially conferring a metabolic resistance to these lipopeptides. The *menH* gene, involved in menaquinone (MQ) biosynthesis, provides a key role in cellular respiratory function and membrane electrochemical potential (Δψ) maintenance [92]. It has been shown that mutants with altered MQ production exhibit increased resistance to lysocin E, a cyclic lipopeptide known to target bacterial MQ in the membrane [123, 124]. However, the effects of excess MQ appear to interfere with lysocin E activity, suggesting competition between the peptide and the coenzyme for membrane binding sites [123]. This suggests a potential indirect mechanism whereby alterations in metabolic compounds may influence brevibacillin activity. Similarly, a genetic mutation in *plsX*, a gene encoding a key phospholipid-synthesizing enzyme, could affect the lipid composition of bacterial cell membranes, affecting both their fluidity and their charge, as well as preventing interaction between the peptide and the bacterial membrane [125]. It has been documented that inactivation of *plsX* in *Staphylococcus aureus* results in decreased susceptibility to several lipophilic compounds, suggesting that membrane composition or permeability has been altered [93]. Furthermore, despite the fact that *crnA* is conventionally associated with creatinine metabolism, it has been proposed that its mutation may result in a reorientation of nitrogen metabolism, inducing a state of stress or cellular inactivation similar to persistent cells and consequently conferring a transitory antimicrobial tolerance and adaptation [126, 127]. Previous studies have shown the impact of mutations in metabolic pathways on the natural selection of genes involved in nitrogen metabolism in *S.aureus*, indicating that these metabolic pathways, particularly nitrogen-related ones, are prone to high mutation frequency, indicating a selective pressure exerted during bacterial colonization [126, 127]. This finding highlights a further in-depth investigation focusing particularly on metabolism-related genes such as *crnA*, as they could influence bacterial adaptability to their ecological niches including antimicrobial exposure, especially in relation to nitrogen uptake. Finally, mutations in the *topA* gene, encoding topoisomerase I, have been reported to induce differential regulation of stress genes and impair bacterial growth [128]. It has been reported that *Escherichia coli*, *topA* mutants exhibit an increased tolerance to various antibacterial molecules via an overall decrease in cellular physiological activity, compromising antimicrobial effectiveness specifically linked with the cellular division process [129]. The exposure to bactericidal AMPs induces a rapid response networks, particularly the SOS response, mainly regulated by the *RecA* and *LexA* proteins [27]. This response enables genotoxic stress toleration through the expression of several DNA repair genes, such as *umuDC*, *dinB* and *recN*, which may promote the accumulation of adaptive mutations [130–132]. In the framework of our genomic investigation, we have identified specific gene mutations potentially related to antimicrobial resistance phenotypes, suggesting an association between stress adaptation responses and the emergence of resistant variants. However, these findings do not demonstrate direct causality, and further functional validation will be required to confirm the individual contribution of each mutation to AMPs resistance acquisition [133, 134]. These findings support the hypothesis that antimicrobial peptides might not exert selective pressure, they might also promote bacterial adaptation through the induction of regulated mutagenesis mechanisms.

While the classic approach focuses on genetic resistance mechanisms, recent scientific studies have also suggested that metabolic pathways also provide a crucial adaptive network enabling bacterial persistence and resistance in hostile environments, particularly under antimicrobial pressure [135, 136]. This exposure may involve major metabolic reprogramming that affect central pathways such as glycolysis, the TCA cycle and amino acid biosynthesis, with the aim of redirecting energy resources to survival mechanisms [27]. However, these adaptive metabolic adjustments could be associated with a fitness cost, expressed as reduced growth or enhanced competitiveness of resistant mutants under selective pressure [137]. This dynamic therefore promotes the emergence of variants able to survive regardless of peptide effects [27]. In our fitness cost analysis, the obtained results highlight multiple impacts on metabolic pathways, as well as different resistance patterns detected under various conditions (pH, osmotic and ionic stress). As an example, vPED and vBA adapted variants displayed an enhanced capacity for the uptake of simple substrates such as D-fructose and citrate. This observation highlight a reinforced activation of the glycolytic and TCA cycle pathways, reflecting selective energy optimization to promote antimicrobial resistance through bacterial cell stasis as indicated by growth dynamics in comparison to the wild-type strain [27, 138]. In addition, other variants, such as the vPLS, are distinguished by a preferential exploitation of organic acids such as succinate and malate, reflecting a metabolic switching to secondary pathways. This energetic flexibility has been described as a key factor in bacterial tolerability, particularly in fluctuating environmental conditions or under prolonged exposure to antimicrobial agents [139]. Furthermore, previous studies have highlighted that metabolic adaptations could modulate virulence-related gene expression, underlining an interconnection between metabolism, pathogenicity and resistance. This relationship is also supported by our recent observations, notably through the identification of a mutation in the transcriptional regulator DegU, known for its role in the control of many virulence genes [27, 140]. Nitrogen metabolism modulation also appears to be a significant aspect of this adaptability. Our data for the vNIZ and vBN variants revealed an increased nitrogen compound consumption, indicating selective metabolic regulation to cope with the AMPs induced stresses. In contrast, lower amino acid uptake observed in other adapted variants could indicate a potential repression of energy-demanding biosynthetic pathways, as already documented in *E. coli* during sublethal antibiotic treatments [141]. This cross-regulation of nitrogen assimilation is also likely to impact peptidoglycan composition as well as membrane surface proteins, modifying cell permeability and antimicrobial susceptibility [142]. However, a frequently neglected determinant of antimicrobial resistance is the importance of osmotic metabolism. Our observations concerning some variants, notably vPLS, have revealed an improved growth rate under saline conditions following their exposure to osmoprotectants. These findings suggest that the ability to maintain osmotic homeostasis provides a key adaptive mechanism for preserving bacterial viability under stress conditions, such as exposure to AMPs [143]. This hypothesis is corroborated by several studies that highlighted the importance of osmoprotectants biosynthesis such as trehalose and glycine betaine in resistance to antimicrobial agents [144–146]. Moreover, among the investigated variants, differential responses to pH stress support the hypothesis of metabolic specialization. While VPED and VNIZ displayed a preferential adaptation to acidic environments, vBN and vPLS exhibited an enhanced growth rate under basic conditions. In particular, the vBA variant is distinguished by a metabolic flexibility enabling efficient adaptation to both pH parameters. This phenotypic variability underscores the hypothesis that an increased metabolic plasticity constitutes a selective advantage in a restrictive environment, promoting a faster and more sustained adaptation to external stresses. These observations are supported by numerous studies underscoring that metabolic plasticity constitutes a fundamental adaptive mechanism enabling bacteria to respond to a wide range of environmental stresses, including those induced by exposure to antimicrobial [135, 144, 145, 147]. This metabolic flexibility is reflected by the ability to perform rapid reprogramming of key cellular functions, including membrane permeability, enzymatic activity and essential metabolite biosynthesis [135, 148]. Such transformations allow bacterial cells to preserve sustainability in adverse environments. Moreover, these metabolic adjustments play an active role in the emergence of cross-resistance, characterized by increased tolerance to several classes of antibiotics, underlining the preponderant role of metabolism in the evolutionary dynamics of bacterial resistance [149, 150].

## Conclusion

In summary, the originality of this study relies on the research approach that combine phenotypic, genomics and metabolic analysis. This combination allowed us to better understand and characterize the phenomena involved in the development of resistance to different AMPs according to their structure and mechanisms of action. This study also allowed us to better study the co-resistance between AMPs as well as cross-resistance between AMPs and antibiotics. All these unique data are extremely important to ensure a more effective and better use of new AMPs in the agrifood, veterinary and medical sectors. The effectiveness of the selected AMPs against *L.monocytogenes* ATCC 19,111, as well as the non-identification of cross-resistance patterns between these molecules and conventional antibiotics provide promising strategic perspectives in the control of several pathogenic bacteria. In particular, a rotational approach using these molecules could provide a more effective approach to limit the emergence of antimicrobial resistance. Furthermore, the metabolic analysis of resistant bacterial variants highlights a critical evolution in our understanding of resistance mechanisms, which cannot be sufficiently explained exclusively by genetic determinants. Rather, bacterial metabolism appears to be a major integrative hub linking genomic to environmental pressures. Indeed, it appears to be playing a crucial role not only as a highly responsive regulatory system for antimicrobial stress, but also as a vector for the establishment of long-lasting adaptive mechanisms. These results point out the necessity of extending research beyond conventional resistance models that emphasize genetic mutations and enzymatic inactivation towards a systematic approach focusing on metabolic plasticity as a key component of bacterial adaptability is increasingly required. Faced with the increasing global antimicrobial resistance threat, the efficient identification of key metabolic pathways involved in bacterial resistance is emerging as a particularly promising strategy for the development of innovative strategies to combat and prevent bacterial infectious disease.

## Supplementary Information

Supplementary Material 1.

## Data Availability

The datasets generated and/or analysed during the current study are available in the NCBI public database under the BioProject PRJNA1320689, sample accessions SAMN51163678 to SAMN51163745.
