# Supplementary material for: Microbiological and genomic approaches to study and characterize co- and cross-resistance to antimicrobial peptides in Listeria monocytogenes ATCC 19,111 variants
Source: BMC Microbiol. 2026 Aug 7;26:956. doi: 10.1186/s12866-026-05467-w (PMC13733129; doi:10.1186/s12866-026-05467-w)
Supplement: Supplementary file 1 [file 12866_2026_5467_MOESM1_ESM.docx]

**Supporting Information**

Omar Fliss,^1,2,3,4^ Louis-David Guay,^2,3,4,5^ Nguyen Phuong Pham,^2,3,6^ Philippe Leprohon,^2,3,6^ Ramzi Guerba,^1,4^ Marc Ouellette,^2,3,6^ Natalie Fava,^7,8^ Franco Pagotto,^7,8^ Eric Biron,^2,3,4,5^ Ismail Fliss, *^,1,3,4^

^1^Département des sciences des aliments, Faculté des sciences de l’agriculture et de l’alimentation, Université Laval, Québec (QC), Canada

^2^Centre de recherche du CHU de Québec-Université Laval, Québec (QC), Canada

^3^Research Center in Infectious Diseases, Université Laval, Québec (QC), Canada

^4^Institute of Nutrition and Functional Foods, Université Laval, Québec (QC), Canada

^5^Faculté de pharmacie, Université Laval and PROTEO, The Quebec Network for Research on Protein Function, Engineering, and Applications, Québec (QC), Canada

^6^Département de microbiologie, infectiologie et immunologie, Faculté de médecine, Université Laval, Québec (QC), Canada

^7^Listeriosis Reference Centre, Microbiology Research Division, Bureau of Microbial and Nutrition Hazards, Food Directorate, Health Canada, Canada.

^8^Microbiology Research Division, Bureau of Microbial Hazards, Food and Nutrition Directorate, Health Canada, Canada.

*Corresponding author: [ismail.fliss @fsaa.ulaval.ca](mailto:eric.biron@pha.ulaval.ca) ; Tel. +1-418-656-2131 ext. 406825

**Fig.S1.** HPLC profiles (λ = 220 nm) and LC-MS spectra of synthesized and purified peptides.

**Brevibacillin**

**Brevibacillin Thr1**

**Fig.S1.** (Continued)

**Nisin Z**

**Pediocin PA-1 (M31L)**

H_2_N-KYYGNGVTCGKHSCSVDWGKATTCIINNGAMAWATGGHQGNHKC-COOH

**Fig.S1.** (Continued

**Plantaricin S (α)**

H_2_N-RNKLAYNMGHYAGKATIFGLAAWALLA-COOH

**Plantaricin S (ß)**

H_2_N-KKKKQSWYAAAGDAIVSFGEGFLNAW-COOH

**Fig.S2.** Agar diffusion assay and antimicrobial susceptibility of L. monocytogenes, ATCC 19111 and HPB 568 against the selected AMPs. The pediocin PA-1 (M31L) (A), brevibacillin (B), and its analog (C) as well as nisin Z (D) and plantaricin S (E). Sterile ultrapure water with 10% DMSO were used as a negative control (T-).

**Fig.S3.** Clustered heatmap showing a comparison of results obtained from carbon utilization assays (PM1).

**Fig.S4.** Clustered heatmap showing a comparison of results obtained from nitrogen utilization assays (PM2).

**Fig.S5.** Clustered heatmap showing a comparison of results obtained from osmotic and ionic response assays (PM9).

**Fig.S6.** Clustered heatmap showing a comparison of results obtained from pH response assays (PM10).

**Table.S1.** Production yields and quantification of the different antimicrobial peptides.

| Peptide | Production | Yield (%) | Purity (%) | LC-MS (m/z) |
| --- | --- | --- | --- | --- |
| Brevibacillin | Chemical synthesis | 14 | 98 | [M+H]⁺ 1584.09  [M+2H]^2^⁺ 792.55 |
| Brevibacillin Thr1 | Chemical synthesis | 18 | 96 | [M+H]⁺ 1602.09  [M+2H]^2^⁺ 801.55 |
| Nisin Z | Salting out | 35 | 92 | [M+H]⁺ 3330.54  [M+2H]^2^⁺ 1665.77 |
| Pediocin PA-1 (M31L) | Chemical synthesis | 45 | 97.6 | [M+H]⁺ 4611.21  [M+2H]^2^⁺ 2306.11 |
| Plantaricin S (α) | Chemical synthesis | 43 | 93 | [M+H]⁺ 2923.48  [M+2H]2⁺ 1462.24 |
| Plantaricin S (ß) | Chemical synthesis | 47 | 94 | [M+H]⁺ 2874.29  [M+2H]^2^⁺ 1437.65 |

**Table.S2.** The MBC/MIC ratios of the selected AMPs.

| HPB | Serotype | Origin | Sample source | PED | BN | BA | NIZ | PLS |
| --- | --- | --- | --- | --- | --- | --- | --- | --- |
| 2 | 1/2a | food | coleslaw | 2 | 2 | 1 | 4 | 1 |
| 3 | 4b | clinical | N/A | 4 | 1 | 1 | 4 | 4 |
| 12 | 1/2a | clinical | human spinal fluid | 4 | 2 | 2 | 2 | 1 |
| 16 | 3a | clinical | N/A | 4 | 2 | 1 | 4 | 2 |
| 23 | 4d | clinical | N/A | 2 | 1 | 2 | 4 | 2 |
| 394 | 1/2b | food | brie cheese | 8 | 1 | 2 | 2 | 2 |
| 399 | 1/2a | clinical | N/A | 2 | 1 | 1 | 8 | 8 |
| 412 | 1/2a | clinical | N/A | 8 | 1 | 1 | 8 | 2 |
| 413 | 1/2a | clinical | N/A | 2 | 1 | 1 | 2 | 16 |
| 568 | 1/2a | food | cheese | 2 | 2 | 1 | 2 | 1 |
| 769 | 4b | clinical | blood | 2 | 2 | 1 | 2 | 2 |
| 774 | 4c | clinical | N/A | 8 | 1 | 2 | 4 | 1 |
| 850 | 4b | clinical | N/A | 2 | 1 | 1 | 1 | 2 |
| 861 | 4b | food | alfalfa tablets | 8 | 2 | 1 | 2 | 2 |
| 1088 | 4d | clinical | N/A | 8 | 1 | 2 | 8 | 2 |
| 1102 | 4b | clinical | N/A | 8 | 1 | 1 | 2 | 2 |
| 1340 | 1/2b | clinical | N/A | 2 | 1 | 1 | 2 | 2 |
| 1836 | 1/2a | clinical | N/A | 2 | 1 | 2 | 8 | 1 |
| 3034 | 4a | clinical | N/A | 4 | 2 | 1 | 4 | 8 |
| 5622 | 1/2a | clinical | blood | 4 | 1 | 1 | 4 | 1 |

Abbreviations: N/A indicates that no sample source has been indicated. PED, BN, BA, NIZ and PLS are respectively pediocin PA-1 (M31L), brevibacillin , brevibacillin Thr 1, nisin Z and plantaricin S, respectively.
